# Fundamental properties and sustainable applications of the natural zeolite clinoptilolite

**DOI:** 10.1007/s11356-024-33656-5

**Published:** 2024-05-23

**Authors:** Nadia Grifasi, Bianca Ziantoni, Debora Fino, Marco Piumetti

**Affiliations:** Department of Applied Science and Technology, Corso Duca Degli Abruzzi, 24, 10129 Turin, Italy

**Keywords:** Natural zeolite clinoptilolite, Sustainable material, Wastewater treatment, Gas purification, Clean thermal energy source, Medical application

## Abstract

**Abstract:**

This review explores a set of sustainable applications of clinoptilolite, a natural zeolite abundant around the world in different localities. Thanks to its physico-chemical properties this material is extremely versatile for several applications, ranging from environmental catalysis and CO_2_ removal to industrial and agricultural wastewater purification, aquaculture, animal feeding, and food industry but also medical applications and energy storage systems. Due to the presence of cations in its framework, it is possible to tune the material’s features making it suitable for adsorbing specific compounds. Thus, this review aims to provide insight into developing new technologies based on the use of this material that is sustainable, not harmful for humans and animals, naturally abundant, and above all cost-effective. Furthermore, it is intended to promote the use of natural materials in various areas with a view to sustainability and to reduce as far as possible the use of chemicals or other materials whose synthesis process can have a polluting effect on the environment.

**Graphical abstract:**

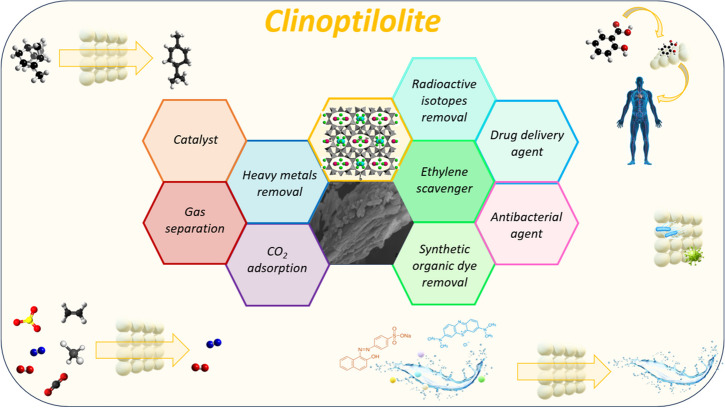

## Introduction

Zeolites are crystalline solids characterized by well-defined three-dimensional structures containing micropores. Both natural and synthetic ones can be classified depending on framework structure and framework type (Das and Das 2020; McCusker and Baerlocher 2001). According to the Commission on New Minerals and Mineral Names (CNMMN), all the zeolites are characterized by a three-dimensional, four-connected anionic scaffold, known as framework *structure*, which is constituted by plenty of corner-sharing TO_4_ tetrahedra, where T represents any tetrahedrally coordinated cation (Piumetti et al. 2022). It results relatively open, with a series of channels and cavities. It should be observed that the framework structure is a crucial parameter for the understanding of zeolite chemistry. Another important element for the classification of all types of zeolites is the framework *type* which, differently from the framework structure, can be defined as a description of the connectivity of the tetrahedrally coordinated atoms of the framework as symmetrically as possible (Katzer 1989; van Koningsveld 2007). Just an example, in the literature, it is reported that the framework type affects the interaction between zeolite and specific molecules. In fact, zeolite types such as MOR and ZSM-5 promote the interaction and affinity to CO_2_ more than USY and BEA zeolites (Bacariza et al. 2019). Considering the framework type, since the composition, the observed symmetry, or the actual unit cell dimensions are neglected, these results are a good way to classify different materials under a single designation. More precisely, all the zeolites are labeled with a three-letter code that identifies the framework type. As previously mentioned, the structural framework of zeolites consists of TO_4_ tetrahedra, specifically [SiO_4_] and [AlO_4_] tetrahedra. The coordination between aluminum and silicon atoms occurs through shared oxygen atoms in a tetrahedral arrangement. Electrostatic forces prevent the formation of an Al-O-Al bond due to the charge repulsion between aluminum atoms. The result of this arrangement gives cages connected by pore openings of a defined size (Breck 1973; Martínez and Corma 2013; Schulman et al. 2020). The negative charge on the lattice is neutralized by the positive charge of the cations located within the material’s pores. Each AlO_4_ tetrahedron in the framework bears a net negative charge which is balanced by additional non-framework cations, mainly alkali and alkaline-earth metals, i.e., Na^+^, K^+^, Ca^2+^, and so on. Moreover, the interconnected cavities also host water molecules; in fact, zeolites are known as hydrated minerals. Both cations and water molecules within the honeycomb-like structure of zeolites can move easily, allowing reversible dehydration and cation exchange. Thus, structural and chemical properties can vary strongly, depending on the size of the cavities and the number of connections between them (Bogdanov et al. 2009; Cundy and Cox 2003). Zeolites are usually synthesized under hydrothermal conditions inside autoclaves or digestors, starting from reactive gels in alkaline media at temperatures between about 80 and 200 °C, emulating the conditions occurring during their natural formation (Cundy and Cox 2003; Favvas et al. 2016; Khaleque et al. 2020; Rosso et al. 2022). The main feature of synthetic zeolites is the regularity of the porous structure and channel size. In fact, controlling the synthesis in the laboratory allows a high degree of reproducibility of the material achieved, and this is generally preferred for industrial catalytic applications aimed at producing certain chemical compounds. However, the high production cost of synthetic zeolites encourages the search for more cost-effective materials (Breck 1973). In fact, it is well known that zeolites can also occur naturally in volcanic rocks due to chemical reactions that could last hundreds or thousands of years. Precisely, more than 50 different natural zeolites have been discovered over the years, and, depending on the origin, different cations are entrapped in the framework, leading to different properties. It is noteworthy that these minerals were already known to mankind over the centuries; in fact, they were used to build pyramids and temples in Mexico or churches and houses in Cappadocia. But it was in the late 1950s that geological discoveries revealed the existence of large sedimentary deposits of natural zeolites in different countries around the World, i.e., Australia, Bulgaria, Canada, Cuba, Georgia, Hungary, Italy, Japan, Mexico, Romania, Russia, Serbia, Slovakia, Turkey, Ukraine, and the USA (Korkuna et al. 2006; Production and Relations 2019; Roque-Malherbe 2001). Table 1 reports some of the most common natural zeolites and their respective unit cell chemical formula (Iijima 2007; Roque-Malherbe 2001).

**Table 1:** List of common natural zeolites. Adapted from (Iijima 2007; Roque-Malherbe 2001)

**Zeolite**	**Framework type**	**Host rocks**	**Unit cell chemical composition**
Analcime	ANA	U, B, I, A	*Na*_16_(*Al*_16_*Si*_32_*O*_96_) ∙ 16*H*_2_*O*
Chabazite	CHA	U, B, I, A	*Ca*_6_(*Al*_12_*Si*_24_*O*_72_) ∙ 40*H*_2_*O*
Dachiardite	DAC	B, A	*Na*_5_(*Al*_5_*Si*_19_*O*_48_) ∙ 12*H*_2_*O*
Erionite	ERI	B, I, A	*NaK*_2_*MgCa*_2_(*Al*_9_*Si*_27_*O*_72_) ∙ 27*H*_2_*O*
Faujasite	FAU	U, B	*Na*_58_(*Al*_58_*Si*_134_*O*_384_) ∙ 240*H*_2_*O*
Heulandite	HEU	B, I, A	*NaCa*_4_(*Al*_9_*Si*_27_*O*_72_) ∙ 24*H*_2_*O*
Clinoptilolite	HEU	B, I, A	*K*_2_*Na*_2_*Ca*(*Al*_6_*Si*_30_*O*_72_) ∙ 21*H*_2_*O*
Mordenite	MOR	B, I, A	*Na*_4_*Ca*_2_(*Al*_8_*Si*_40_*O*_90_) ∙ 24*H*_2_*O*
Philipsite	PHI	U, B, I, A	*K*_2_*CaNa*_2_(*Al*_5_*Si*_11_*O*_32_) ∙ 12*H*_2_*O*
Stilbite	STI	B, I, A	*Na*_4_*Ca*_8_(*Al*_20_*Si*_52_*O*_144_) ∙ 56*H*_2_*O*

U➔ ultrabasic rock

B ➔ basaltic rock

I ➔ intermediate rock.

A ➔ acidic rock

**Fig. 1:**
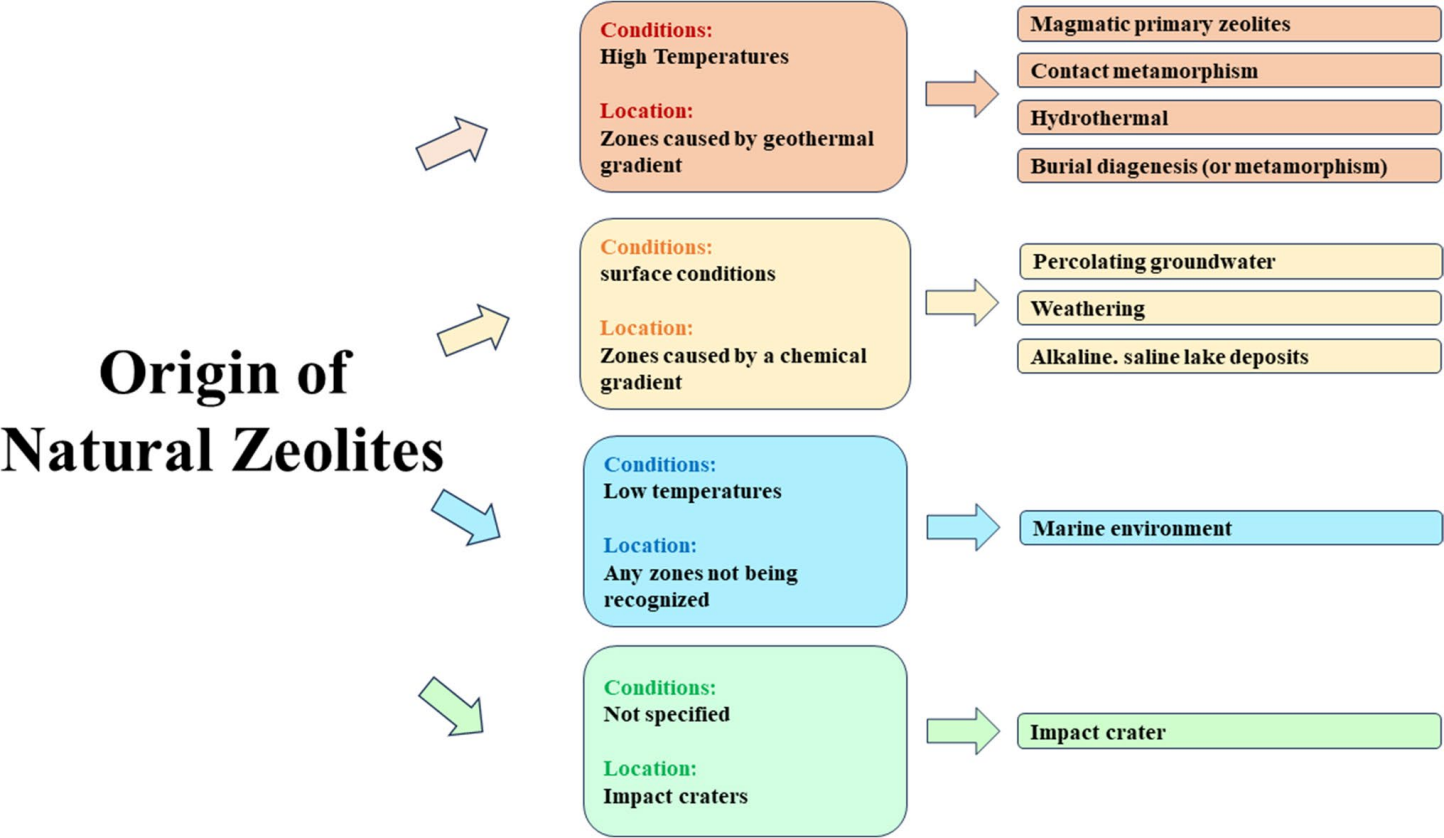
Schematization of the origin of natural zeolites. Adapted from (Iijima 2007)

Typically, natural zeolites exhibit a limited crystallinity compared to synthetic ones, due to a certain degree of contamination from minerals present in the structure or other impurities. This is the main reason that prevents their current massive use in the industrial sector (i.e., as catalysts). Nevertheless, both physical and chemical modifications can be adopted to improve their quality (Favvas et al. 2016; Hernández et al. 2000). One successful method for modification consists of the use of acids. The feasibility of acid treatments on natural zeolites is due to their acid stability, for example in the presence of sulfuric acid, hydrochloric acid, nitric acid, or acetic acid. The main aim of these treatments is to remove the aluminum from the aluminosilicate framework thus increasing the Si/Al ratio and, consequently, creating more void spaces (pores) within the material, increasing the surface area and improving the adsorption efficiency (Mansouri et al. 2013; Silva et al. 2019). Moreover, some studies revealed that natural zeolites show excellent pozzolanic properties like resistance to sulfate attacks, alkali-aggregate reactions, chloride diffusion, and shrinkage strains. This makes them the ideal candidates to substitute pozzolan in cement manufacture, in case of shortage of this material (Bilim 2011; Kriptavičius et al. 2022). Naturally, clinoptilolite-rich zeolites exhibit the intrinsic property of readily reacting with lime. Thanks to this reaction, the long-term strength, as well as the refinement of the pore structure of the blended concrete, is enhanced. Regarding cost analysis, it should be emphasized that commercial zeolites can be synthesized from different raw materials which can be natural or synthetic as well, but not all of them are equally convenient from an economic point of view (Rosso et al. 2022). The average cost of commercial synthetic zeolites can fluctuate according to both the quantity purchased and the supplier. Moreover, even the process of synthesis can have an impact on the final price. In fact, synthetic zeolites can be obtained from both physicochemical and solvothermal processes, including sol-gel, hydrothermal, alkali fusion, and alkali leaching methods (Khaleque et al. 2020). Hydrothermal technique, which involves the use of water as a solvent and a base as a mineralizer for the production of commercial zeolites, provides a good quality product but it involves the use of very expensive autoclaves, and it requires the cost of the disposal of corrosive slurries produced (Rabenau 1985; Sugano et al. 2005). On the other hand, the sol-gel process involves the formation of an inorganic colloidal suspension (sol) that undergoes a gelation step thus forming a continuous liquid phase (gel) that later turns into a three-dimensional network structure. The advantage of this method consists in better control of the technique which thus leads to higher porosity and definite particle size. However, the main economic disadvantage relies on the cost of the precursor (Hench and West 1990). Besides, the synthesis of zeolites through the alkali fusion technique is very efficient, but it requires high pressures and temperature, thus needing a huge energy consumption and high associated costs. Finally, alkali leaching is another synthesis method characterized by the use of leachates such as NaOH solutions. This method has been exploited to obtain synthetic zeolites from fly ashes (El-Naggar et al. 2008). However, this is a multistep process and consequently it requires a not negligible amount of time and expense. Having made these assumptions, natural zeolites represent abundant and low-cost natural minerals. They predominantly occur in proximity to volcanic areas, although their formation is not limited to specific environments and can take place under varied physico-chemical conditions. The temperature and chemical composition of the host rocks play pivotal roles in determining the characteristics of the resulting natural zeolite. These zeolites can be categorized into basaltic, intermediate, and acidic types (refer to Table 1) based on these conditions (Iijima 2007). Additionally, the pH, alkalinity, and concentration of chemical components are crucial factors influencing zeolite formation, particularly in saline lake deposits. Deposits have been identified in diverse settings, including mafic volcanic rocks (deposited by fluids or vapors), sedimentary rocks (as alteration products of volcanic glass), acidic or basic volcanic glass, clay, detrital rocks, and sedimentary rocks across various oceans (Borsatto and Inglezakis 2012; IZA Commission on Natural Zeolites n.d.). A schematic representation of the different origins of natural zeolites is reported in Fig. 1.

However, the price of the natural zeolites depends on the origin country, the deposit, the composition, the size of a powder, and the market development (Borsatto and Inglezakis 2012). For these reasons, a precise evaluation of price is very difficult; however, an estimation of the cost could be made considering, i.e., the size of the powder which determines the field of application. For example, the price of clinoptilolite powder ranges from 1.04 €/kg to 22 €/kg if a finer powder is needed (particle size of approximately 200 μm). From literature, it can be found that the cost of natural zeolites containing a huge percentage of clinoptilolite (about 71%) has been estimated at around 200–400 €/ton whereas zeolite 13X has a cost of 2000–3000 €/ton (Jänchen et al. 2015). In the USA, the price can vary from 20 to 100$/ton (200$/ton in the case of clinoptilolite), whereas a synthetic zeolite A ranges from 500 to 600$/ton (Borsatto and Inglezakis 2012). It should be mentioned that market data related to annual sales of synthetic zeolites have recorded an average cost of 2750$–3000$ USD per ton, and this price was estimated on the basis of 150,000–200,000 tons sold up to 2015 that generated an income of approximately 450$–500$ million USD (Kouchachvili et al. 2023). Thus, it is evident the economic advantage of using natural zeolites compared to the synthetic ones. Among natural zeolites, clinoptilolite has received great attention over the decades, due to its chemical stability in various caustic media, thermostability, high rate of sorption equilibrium, and so on, which promote its use in the environmental applications and industry (Bogdanov et al. 2009). This review aims to explore the diverse applications of clinoptilolite across various fields. Its exceptional versatility opens up a wide range of possibilities, including environmental catalysis, CO_2_ removal, industrial and agricultural wastewater purification, aquaculture, animal feeding, the food industry, medical applications, and energy storage systems. The presence of cations in its framework allows for the fine-tuning of features, making it adept at adsorbing specific compounds. The primary objective of this review is to offer insights into the development of sustainable technologies utilizing clinoptilolite. The definition of “sustainability” is very complex and implies keeping in mind different aspects. One meaning of “sustainability” was found in the report of the Brundtland Commission of the United Nations, which defined it as “the development that meets the needs of the present without compromising the ability of future generations to meet their own needs” (Bartlett 2012; Ehnert 2009). This definition leads generations towards the possibility of using the planet’s resources and has become one of the most quoted definitions. Sustainability presupposes that resources are limited and must be used conservatively and wisely, taking into account the priorities and long-term consequences of how resources are used. One key dimension concerns the relationship between the environment and humans. The World Conservation Strategy defined “sustainable development” in terms of both improvements in human life and conservation of natural resources. Moreover, social and economic responsibility should be also considered (Busco et al. 2013; Giovannoni and Fabietti 2013). Based on these considerations, this review aims to provide an insight into developing new technologies based on the use of this natural zeolite that is sustainable, not harmful for humans and animals, naturally abundant, and above all cost-effective. Furthermore, it is intended to promote the use of natural clinoptilolite in various areas with a view to sustainability and to reduce as far as possible the use of chemicals or other materials, thus minimizing environmental impact and leveraging natural resources. Thus, this material, being naturally abundant, cost-effective, and non-harmful to humans and animals, holds promise for innovative and eco-friendly applications.

## Natural origin, structure, and main properties of clinoptilolite

### Formation process in nature

Clinoptilolite is commonly found in Paleogene and Cretaceous calcareous sediments, as well as terrigenous clay, in the Atlantic Ocean and the Pacific Margin. It is also present in other sediments in the Pacific and Indian Oceans. Typically, this mineral is associated with low-temperature environments (60–110 °C), resulting from the alteration of volcanic rocks. This alteration occurs due to a reaction between acidic volcanic glass and interstitial solutions during burial diagenesis (Iijima 2007). In areas with slow sedimentation, such as hydrologically closed systems, the presence of clinoptilolite is attributed to the reaction between basaltic glass and water containing high levels of dissolved silica, high salinity, and high pH. The resulting zeolite tends to be nearly pure, contributing to its substantial economic value.

The Commission of Natural Zeolite (IZA) (IZA Commission on Natural Zeolites n.d.) has extensively documented various origins of clinoptilolite, one of which involves its formation through the diagenesis of marine pyroclastic and volcaniclastic sequences. In this scenario, zeolite emerges from solutions comprising marine water heated by the anomalous geothermal gradient in active volcanic areas and/or by hot pyroclastic rocks. The resulting zeolites are categorized as clinoptilolite-Na, clinoptilolite-Ca, and clinoptilolite-K. Clinoptilolite also originates in calcareous soils developed on tuffaceous sediment, with notable deposits discovered in locations such as Texas, Hungary, Bulgaria, Romania, Lebanon, India, Japan, and Sicily. Furthermore, clinoptilolite is found in deep-sea sediment, particularly in the core ocean basins far removed from continents. This is particularly true for the k-series of clinoptilolite (IZA Commission on Natural Zeolites n.d.; Rodríguez-Iznaga et al. 2022).

### Morphology, structure, and textural properties of clinoptilolite

The name clinoptilolite derives from the Greek words κλίνω, meaning oblique, *φτερών*, which means feather and λίθος that is stone, thus resulting “oblique feather stone.” This name is due to the reason for which it was believed that it was the monoclinic (or obliquely inclined) phase of the mineral “ptilolite.” Subsequently, it was revealed that “ptilolite” was the former name of the mineral mordenite, rendering it obsolete (Bilgin 2017; Tzia and Zorpas 2012).

**Fig. 2:**
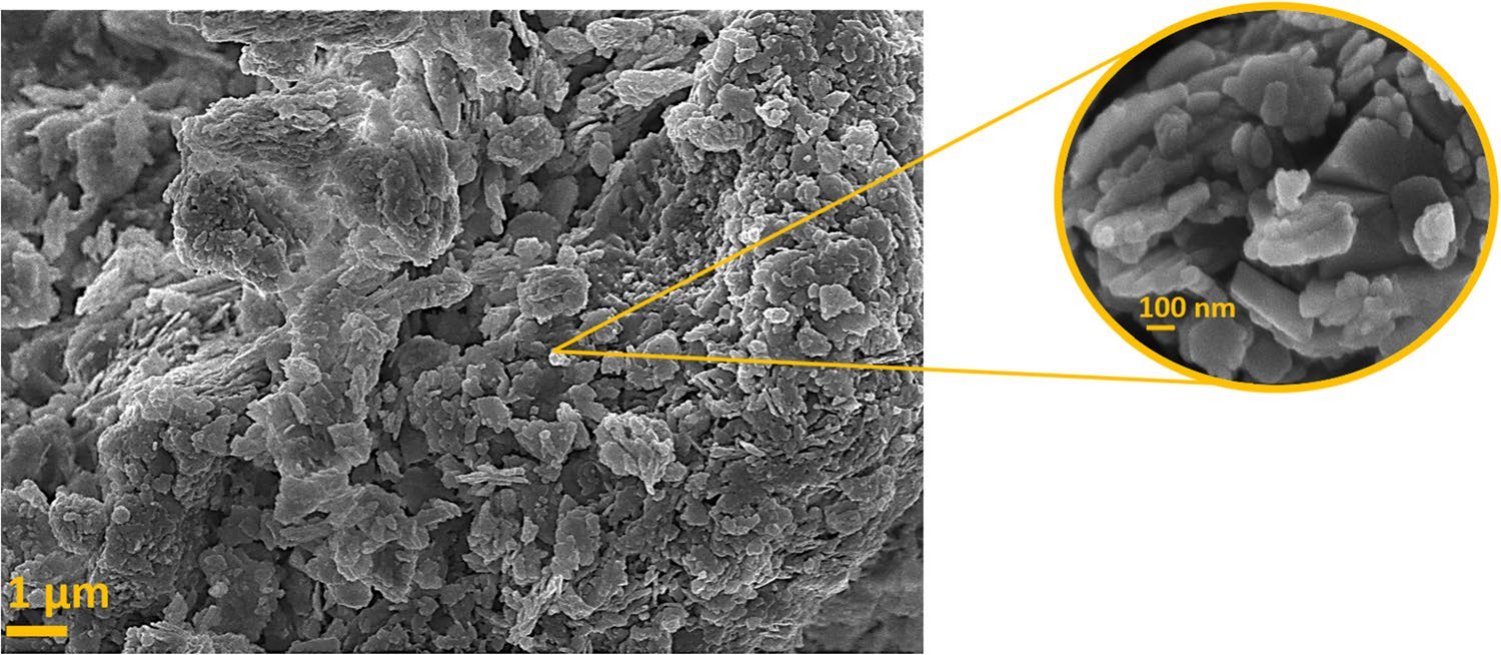
FESEM micrographs of clinoptilolite (from a deposit in Greece) acquired at 1 μm (left) 100 nm (right) magnifications

Even though nowadays clinoptilolite is considered the most common and abundant natural zeolite, it was not described as an individual mineral species until 1932, when Scheler proposed the name clinoptilolite because of the chemical similarity between clinoptilolite and mordenite (ptilolite). Clinoptilolite can be usually found in some sedimentary deposits in association with Heulandite. It is also known that the relationship between clinoptilolite and heulandite originated historically because of the misconnection of clinoptilolite itself (monoclinic) and mordenite or ptilolite (characterized by aggregates of needles with parallel extinction), which led to incorrect divergence between clinoptilolite and heulandite species before their framework structures resulted to be identical. Later, in 1934, Hey and Bannister concluded that clinoptilolite was simply a silica-rich heulandite, according to similarities in their X-ray diffraction patterns (Bish and Boak 2001). Clinoptilolite possesses the same tetrahedral framework as heulandite (named HEU), exhibiting a sheet-like structural organization. By performing a FESEM analysis, it is possible to see the typical “flake” structure of the clinoptilolite, as reported in Fig. 2. The particles appeared aggregated and characterized by grains with no well-defined crystal faces, as observed by different studies (Dosa et al. 2022; Galletti et al. 2021; Szymaszek-Wawryca et al. 2022)

**Fig. 3:**
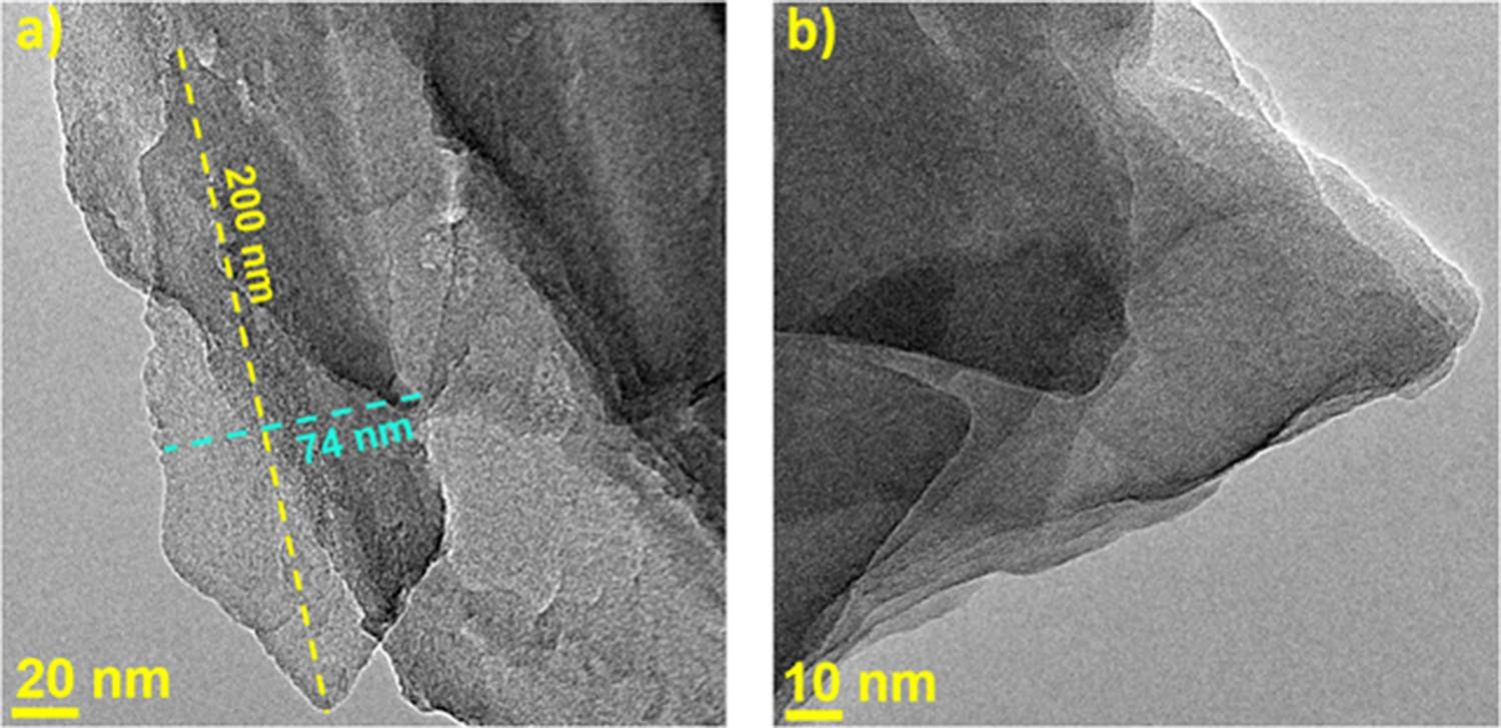
TEM images of clinoptilolite (from a deposit in Greece) at two different magnifications, 20 nm (**a**) and 10 nm (**b**). Adapted from (Dosa et al. 2022)

On the other hand, TEM analysis could give additional information about the structure and the dimension of a specific particle evaluated through the use of the Software Image J, as reported in Fig. 3 (Dosa et al. 2022). The images indicate that Clinoptilolite particles are relatively large, displaying a “plate”-like structural arrangement, with an average dimension of approximately 200 × 74 nm.

This mineral contains cavities filled with water molecules and different cations. The water molecules can be easily removed by thermal treatments in the air without significant changes of the framework, whereas the cations are usually exchangeable at low-mild temperatures (namely, below 100°C).

**Fig. 4:**
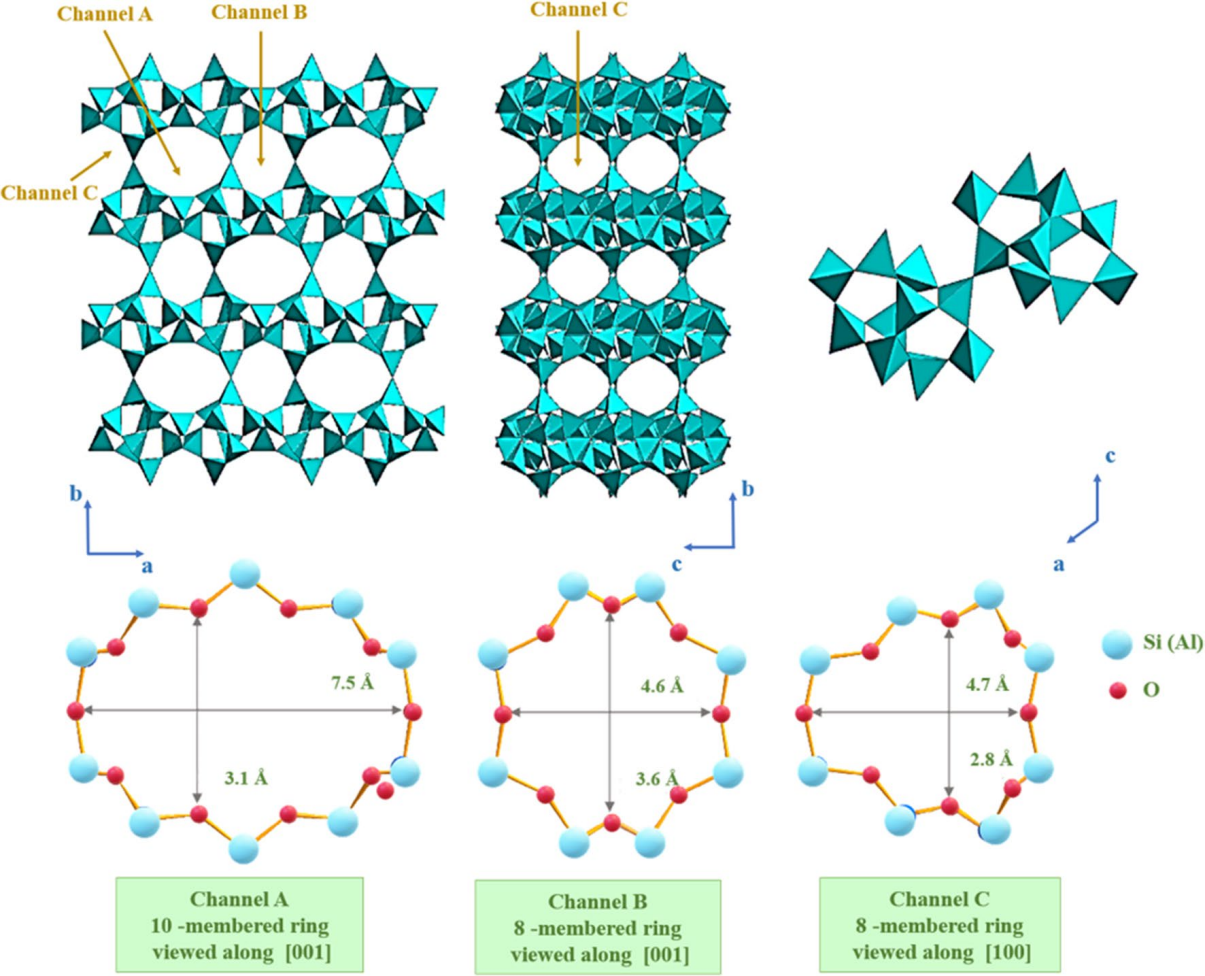
Schematic representation of the clinoptilolite structure. Adapted from (Rodríguez-Iznaga et al. 2022)

The crystalline structure of clinoptilolite consists of two-dimensional intersecting channels, named channels A (10-membered ring) with size 3.1×7.5 Å and B (8-membered ring) with size 3.6×4.6 Å, which are parallel to each other, and another channel, named channel C (8-membered ring) with size 2.8×4.7 Å which intersects both A and B. A schematic representation is reported in Fig. 4. Rings belonging to different sheets are connected throughout the crystal structure by channels whose dimensions determine the size of molecules or ions that can cross the internal framework (Tzia and Zorpas 2012).

**Fig. 5:**
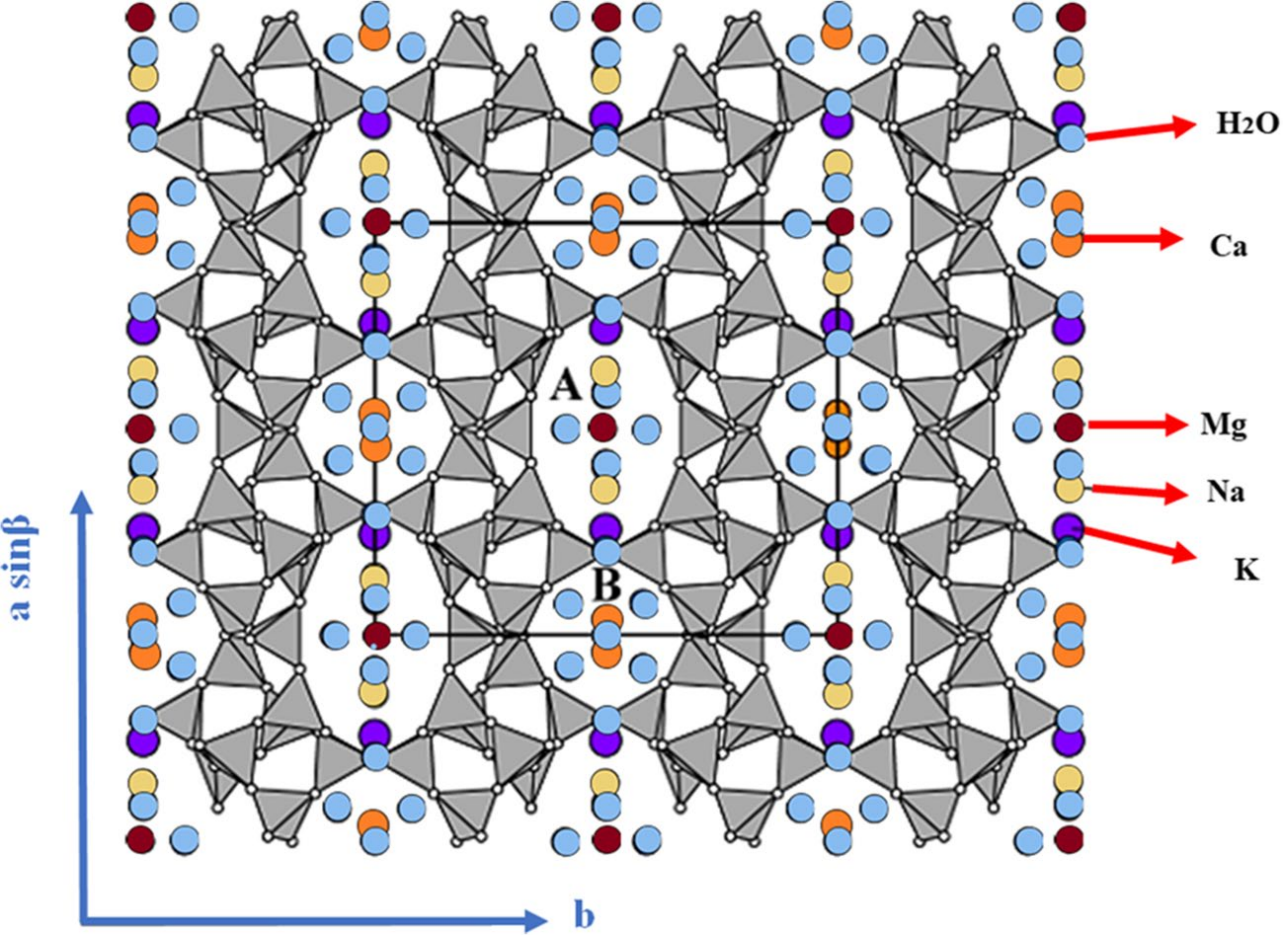
Representation of cations and water molecules into the clinoptilolite framework. Adapted from IZA Commission on Natural Zeolites (n.d.)

These channels are occupied with specific cations, mainly alkaline or alkaline-earth metals, which compensate the Al-induced charge imbalance, as reported in Fig. 5. The general chemical formula of clinoptilolite is *K*_2_*Na*_2_*Ca*(*Al*_6_*Si*_30_*O*_72_) ∙ 21*H*_2_*O* (for unit cell). The H_2_O molecules occurring in channel B are commonly fully occupied, whereas those occurring in channel A are generally only partially occupied (IZA Commission on Natural Zeolites n.d.). Depending on the nature and the dimension of the cations, they can occupy different positions in the framework; i.e., Na^+^ and Ca^2+^ can be located both in the 10-member ring channels A and in the 8-member ring channels B. Moreover, Na^+^ is nine-coordinated with four framework oxygens and five strongly disordered and partially occupied H_2_O molecules, whereas cation Ca^2+^ is eight-coordinated with four framework oxygens and four-channel H_2_O molecules. Cation K^+^ can be located in the 8-member ring vertical channel C, exhibiting the same coordination as Ca^2+^ and cation Mg^2+^ commonly residing in the center of the channel of 10-member rings (channel A), coordinated only to six disordered H_2_O molecules (Ackley et al. 1992; IZA Commission on Natural Zeolites n.d.; Favvas et al. 2016). Thus, it is clear that clinoptilolite channels display a selectivity toward certain cations (Bogdanov et al. 2009).

**Fig. 6:**
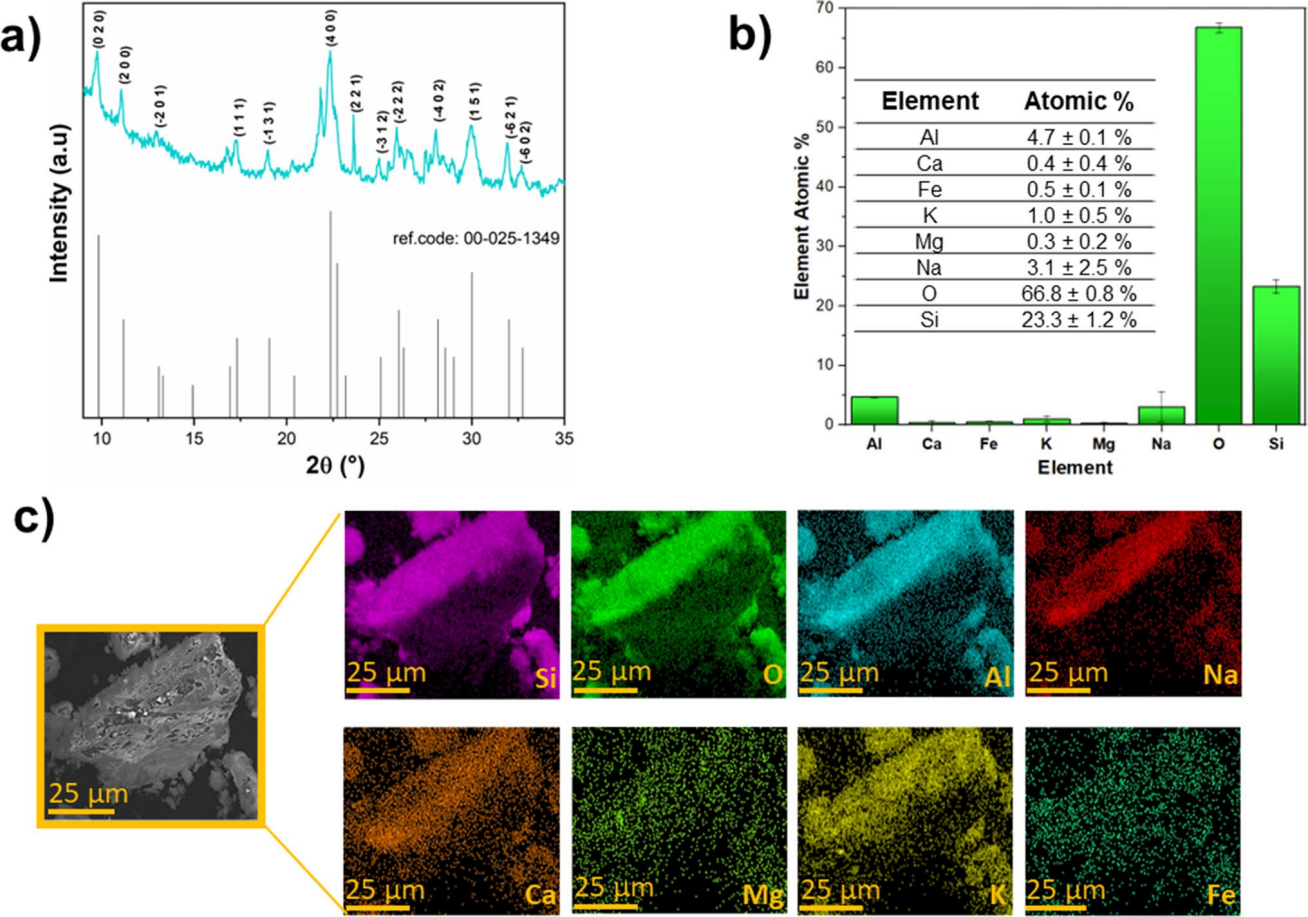
XRD pattern (**a**) and chemical composition (**b**, **c**) performed by EDX analysis of clinoptilolite (from a deposit in England)

As previously mentioned, three different types of clinoptilolite may occur around the world. Clinoptilolite-K is the most widespread, and most dominant in deep-sea deposits. Both clinoptilolite-Na and clinoptilolite-Ca occur in a wide range of environments, including diagenetic replacement of rhyolitic volcaniclastic rocks, active hydrothermal systems, and fractures and cavities in volcanic rocks. Mg is present in almost all clinoptilolites, but higher amounts (above 1 wt%) are found in zeolites originating from volcanic rocks. Fe is mainly present as Fe^3+^ and resides in tetrahedral sites but amounts over 0.5 atoms/cell may be from included hematite. The presence of Sr and Ba is less common in clinoptilolite than in heulandite, but they could be found (IZA Commission on Natural Zeolites n.d.). For example, in Fig. 6, we reported the elemental composition of a clinoptilolite from a deposit in England estimated through EDX analysis.

As can be seen, this clinoptilolite contains several elements in the framework which are well distributed. As expected, the main elements are Si, O, and Al; other cations (i.e., Fe, Ca, Mg, and Ca) are present in smaller quantities as if they were impurities. These values were compared with those of a clinoptilolite from a deposit in Greece obtained in previous work (Dosa et al. 2018), and a good congruence was obtained. The small variations in percentage may be ascribed to the different geographical areas in which the deposit is located.

Although both clinoptilolite and heulandite exhibit similar frameworks, it is possible to distinguish them considering different parameters, i.e., the Si/Al ratio, which is always higher than four for the former and always lower than four for the latter (Gottardi et al. 1985; Radosavljevic-Mihajlovic 2003; Rodríguez-Iznaga et al. 2022). In other words, clinoptilolite is different from heulandite because of its higher content of silicon and, consequently, higher thermal stability (Bilgin 2017). There is also another distinction based on the cationic content since while clinoptilolite is rich in alkali metals such as Li^+^, Na^+^, and K^+^, in the Heulandite the presence of alkaline earth metals (Mg^2+^, Ca^2+^, Ba^2+^) is predominant (Gottardi et al. 1985; Mason and Sand 1960). This is attributed to the different Si/Al ratios (see later). Finally, clinoptilolite and heulandite differ in terms of H_2_O content which is a function of their non-framework cation chemistry and hydration state (IZA Commission on Natural Zeolites n.d.).

Typically, natural clinoptilolites are characterized by small specific surface areas. In fact, performing a N_2_-physisorption at −196 °C on a sample of clinoptilolite (from a deposit in England), it was found that the specific surface area is about 26 m^2^/g (BET method). This result is in agreement with those obtained with other clinoptilolites found in different geographical areas (i.e., 35 m^2^/g (Shen et al. 2020), 13 m^2^/g (Szymaszek-Wawryca et al. 2022), 22 m^2^/g (Cavallo et al. 2023), and so on). Moreover, by comparing the specific surface area of different natural zeolites, except for Chabazite, it is clear that all these minerals have quite low surface area values of between 10 and 30 m^2^/g (see Table 2), and this is in agreement with the above.

**Table 2:** Specific surface area (m^2^/g), mean pore diameter (nm), and pore volume (cm^3^/g) of different natural zeolites

**Zeolite**	**Specific surface area** **[m**^**2**^**/g]**	**Mean pore diameter [nm]**	**Total pore volume [cm**^**3**^**/g]**	**Micropore Volume** **[cm**^**3**^**/g]**	**Ref.**
Clinoptilolite	27	7.0	0.136	6.50 10^−4^	Shen et al. (2020); Velarde et al. (2024)
Chabazite	202	2.3–3.1	0.150	–	Aysan et al. (2016); Leyva-Ramos et al. (2010)
Phillipsite	20	3.3–4.8	0.010	3.21 10^−3^	García et al. (1993); Notario et al. (1995)
Mordenite	14	3.1	0.053	1.00 10^−3^	Korkuna et al. (2006)
Heulandite	13	17.2	0.089	6.47 10^−3^	Tsitsishvili et al. (2023)

**Fig. 7:**
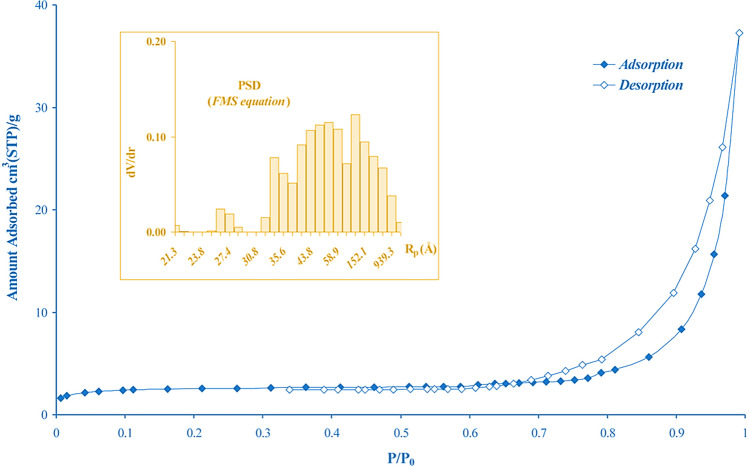
N_2_ adsorption-desorption isotherm and pore size distribution of clinoptilolite. Adapted from (Favvas et al. 2016) with the permission of Elsevier

Figure 7 shows the N_2_ adsorption-desorption isotherm, while the inset describes the pore size distribution according to the FMS equation and using the desorption branch of the isotherm (Favvas et al. 2016).

The isotherm showed a hysteresis loop in the range of 0.6–1 P/P0 and the shape belongs to a type IV B or type H3 isotherm, according to the IUPAC classification (Alothman 2012). The presence of the hysteresis loop can be attributed to multilayer adsorption and capillary condensation in the mesopores of impurities or in the space between zeolite particles. Furthermore, the pore size distribution reveals that the average pore radius varies between 30 and 150 Å; however, there are also smaller and larger pores (Rp ≈ 1000 Å, Rp < 30 Å).

However, these relatively low values could be due to the difficulty of N_2_ to enter the internal pores because of the presence of exchangeable in the micropores which hinders the diffusion of nitrogen into the inner structure of the material, and whose presence was detected by EDX analysis (see Fig. 6). For a better assessment of the porosity of this microporous material, it is possible to use CO_2_ at 273 K rather than N_2_. The synergistic effect between its slightly lower kinetic diameter (3.3 Å) and the higher temperature of adsorption allows an easier CO_2_ entrance and diffusion into the micropores of the zeolite, thus resulting in higher values of specific surface area, namely, 22 m^2^/g and 240 m^2^/g for N_2_ and CO_2_ physisorption, respectively (Cavallo et al. 2023). Noteworthy, the XR-diffractogram of the clinoptilolite (Fig. 6a) exhibits high crystallinity, with the most intense peaks at 2θ = 8.99°, 11.18°, and 22.37°, according to the reference pattern in the database (00-025-1349). Specifically, the peak at 2θ = 22.37° is the most intense and is denoted by the (4 0 0)-type plane. Davarpanah et al. (2020) demonstrated that the Greek zeolite had different percentages of minerals, i.e., an amorphous phase, kaolinite, illite, and clinoptilolite minerals. The slight difference with that reported in Fig. 6 (which came from a deposit in England) suggests the missing of a few phases, in particular quartz which was not detected in the latter, confirming the fact that origin and geographic deposition also play a role in the presence of crystalline phases in the structure.

### Physico-chemical properties

The physico-chemical properties of zeolites are crucial factors that make them extremely versatile and applicable in various fields. Examples are their porous structure, which is crucial for adsorption and ion exchange, or their molecular sieving effect, which consists of their ability to selectively let molecules of specific sizes and shapes into their pores, while excluding larger or smaller molecules. Another key property is their acidic nature, which makes them valuable as solid acid catalysts in various chemical reactions, especially in the petrochemical field. Hydrophilicity and dehydration properties are important in applications related to water adsorption and purification, and so on. In summary, zeolites possess a unique combination of physical and chemical properties that make them versatile materials in applications ranging from water purification and wastewater treatment to catalysis, gas separation, and ion exchange.

It should be emphasized that the huge variety of application fields for Clinoptilolite is a direct result of its main properties such as high resistance to extreme temperatures and ion-exchange, not to mention the chemically neutral basic structural organization and zeodratation properties.

In the following paragraphs, a more detailed description of the main physico-chemical properties is reported.

#### Thermal stability

The thermal behavior of natural zeolites, precisely of clinoptilolite, is fundamental for their characterization. As previously anticipated, this parameter is strictly connected with the Si/Al ratio. In this context, Lowenstein’s law is helpful to comprehend the relationship between silica and aluminum in the SiO_2_ tetrahedron. According to this rule, Al-O-Al is not possible since it is characterized by the proximity of two adjacent Lowenstein’s charges that would disintegrate the lattice. For this reason, the Si/Al ratio must be greater than 1 (Weitkamp 2000). Moreover, a higher content of Si causes greater thermal stability because of the major strength of the Si-Al bond (with respect to the Al-O one), and a low content of Al (high Si/Al ratio) can be interpreted as an index of higher hydrophilic nature of the material (Rodríguez-Iznaga et al. 2022). The thermal behavior is determined by different parameters which involve complex interactions between framework, cations, and water molecules present in channels. There are different works that studied the thermal properties of zeolites (Rodríguez-Iznaga et al. 2022). Alberti and Vezzalini (1984) classified zeolites into three different categories, according to the changes in frameworks during heat treatment and this subdivision is completely in agreement with the one proposed by Bish and Carey in 2001 (Bish and Carey 2001):

- **Category I:** reversible dehydration process upon heating which is accompanied by a rearrangement of extra-framework cations as well as residual water molecules that do not cause important changes in framework or cell volume.
- **Category II:** reversible dehydration process as in the previous case, but the framework is subject to important distortion as well as a contraction of the cell volume can be seen.
- **Category III:** dehydration process is followed by the destruction of T-O-T bridges (where T can be a Si or Al atom) and thus substantial modifications in framework topology.

**Fig. 8:**
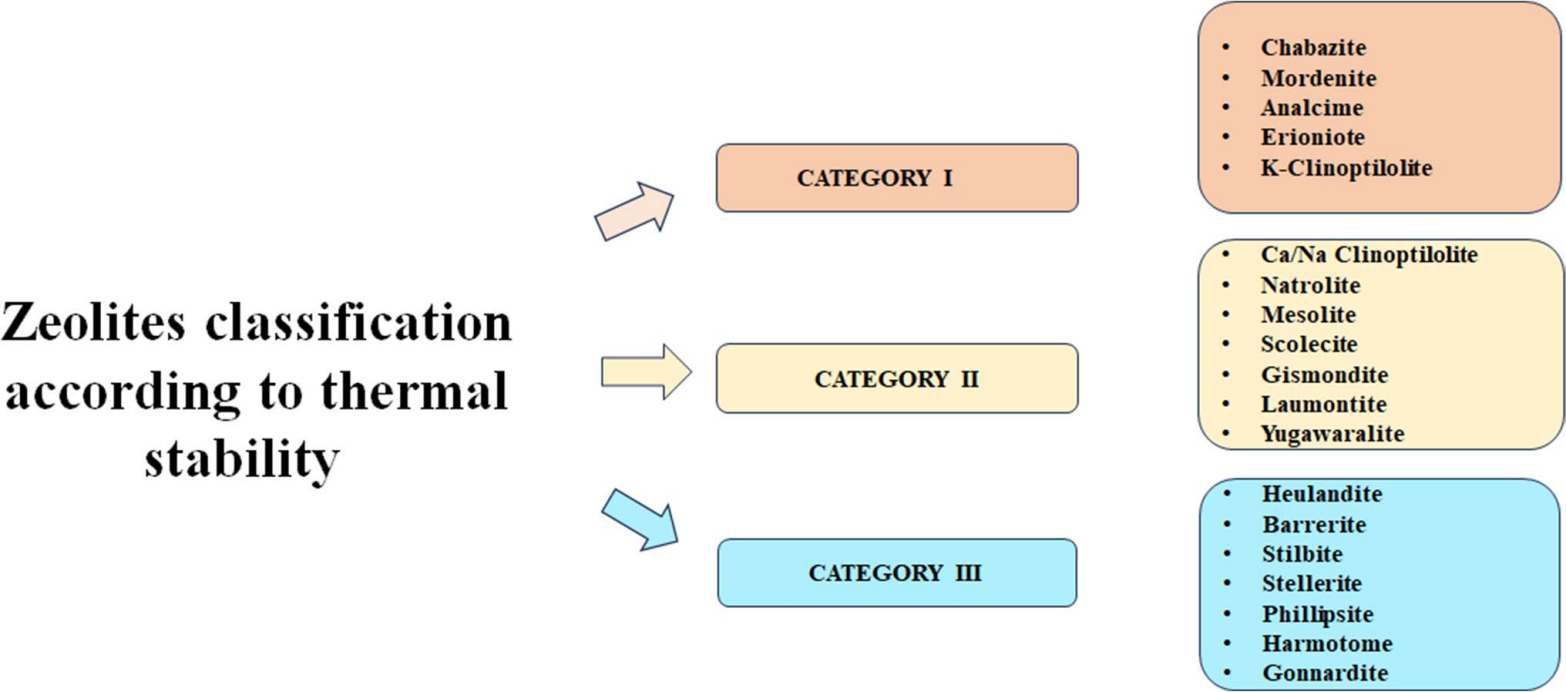
Thermal classification of zeolites (Bish and Carey 2001; Rodríguez-Iznaga et al. 2022)

It is interesting to highlight that a zeolite may belong to different categories, according to the nature of the tetrahedral framework, framework’s charges, and Al/Si ratio as well as type and quantity of extra-framework cations (Bish and Carey 2001), as illustrated in Fig. 8. For example, zeolites belonging to the heulandite family (HEU) such as Heulandite, clinoptilolite, stilbite, barrerite, and stellerite fit to the third category. In fact, when natural heulandite is heated at temperatures in the range between 250 and 500 °C, it moves to the so-called B phase and the rupture of the T-O-T bridges in the four-ring of the cage occurs. However, a clinoptilolite functionalized with ionic exchange can belong also to the categories I and II, depending on the type of extra-framework cation linked to the cell. As a result, K-clinoptilolite can be found in category I whereas clinoptilolite exchanged with Na or Ca totally fits category II.

**Fig. 9:**
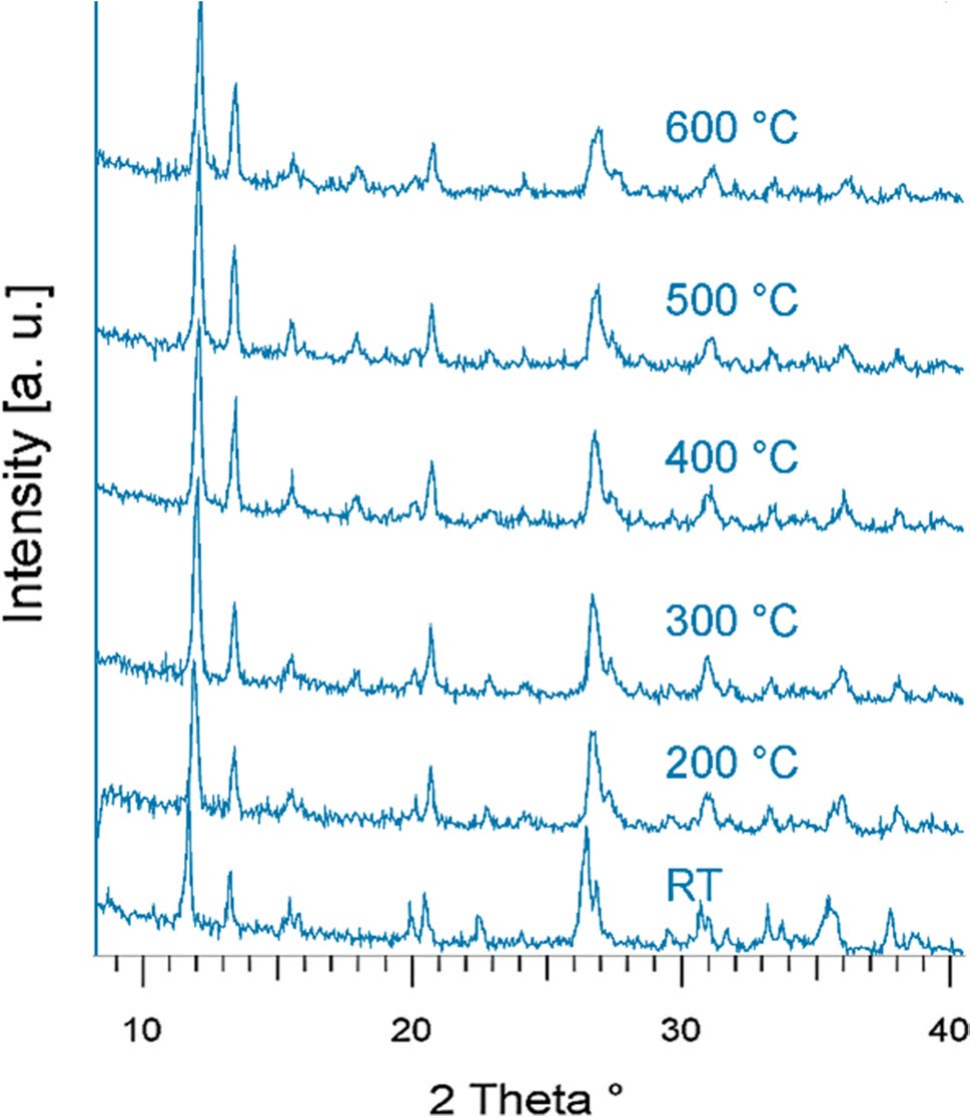
XRD powder patterns of in situ heated sample of clinoptilolite. Image adapted from Dimowa et al. (2013)

Based on the previous considerations, it was demonstrated that when heating up to about 450 °C, the structure of heulandite is destroyed, transforming into “Heulandite B” at 230 °C and becoming amorphous at 350 °C (Boles 1972; Mumpton 1960), whereas the clinoptilolite structure persists stably up to about 700 °C. This behavior was explained by Koyama and Takeuchi (1977) who ascribed the thermal stability to the difference in the Al/Si ratio and to the valency of exchangeable cations. Precisely, the position of potassium atoms plays a role in preventing the ring from collapsing when water molecules are removed. Therefore, since the major cations of the clinoptilolite are monovalent while those of the heulandite are bivalent, this explains the different thermal behavior and categories. Moreover, Dimowa et al. (2013) carried out high-temperature X-ray diffraction analysis on a clinoptilolite taken from a Bulgarian deposit, reporting that no significant structural changes were observed up to 600 °C (see Fig. 9).

**Fig. 10:**
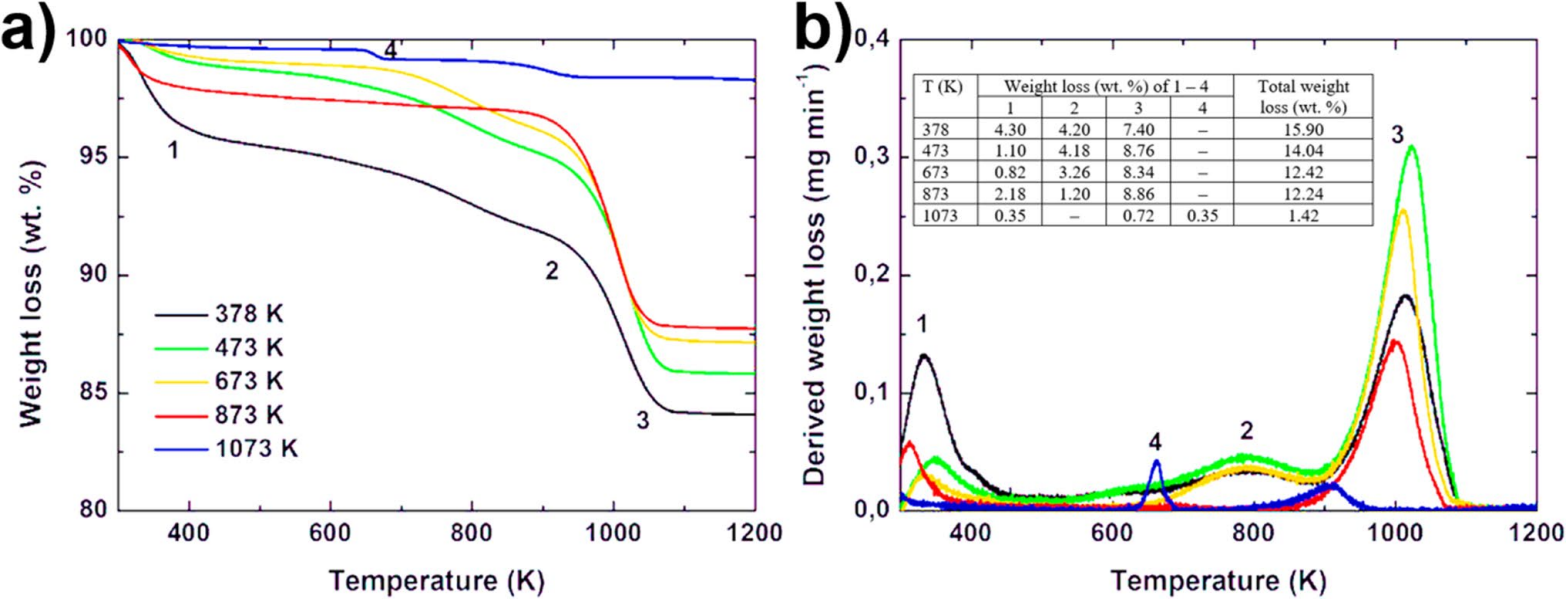
TGA curves (**a**) and DTG profiles (**b**) of clinoptilolite pretreated at 378 K, 473 K, 673 K, 873 K, and 1073 K. Image adapted from Kukobat et al. (2022) with the permission of Elsevier

Moreover, the behavior of clinoptilolite at high temperatures was also investigated by Alver et al. (2010) performing thermogravimetric analyses (TGA and DTG) in the temperature range 30–1000 °C. They observed that all clinoptilolite samples had major, rapid mass losses between 30 °C and 200 °C, with slower and less significant mass losses at higher temperatures. This result was confirmed by Kukobat et al. (2022) performing TGA analyses considering different pretreatment temperatures at which clinoptilolite had undergone, and it was possible to distinguish different profiles (see Fig. 10).

The authors noted that clinoptilolite pretreated at 378 K showed three significant weight losses in the temperature range of 300–1200 K. The first one at 350 K ascribed to the evaporation of externally adsorbed water, and the other two at 800 K and 1000 K referred to the in-pore water evaporation. The same profiles were also observed for the sample treated at 473 and 673 K. On the contrary, by increasing the temperature of the pretreatment (i.e., 1073 K), the profile started to change, and new weight loss appeared at 660 K. From the DTG, it was noted that the clinoptilolite that had undergone high thermal treatment presented smaller intensities and smaller weight loss (ca. 1.42 wt% at 1073 K), compared to that at 378 K (weight loss of 15.90 wt%) suggesting that the externally adsorbed water and in-pore water were significantly removed by treatment.

#### Si/Al ratio and the related properties

The Si/Al ratio is an important parameter that affects the properties of the zeolites, i.e., cation exchange capacity, hydrophobicity, etc… Some values for different natural zeolites are reported in Table 3.

**Table 3:** Si/Al ratio, CEC, and exchange selectivity of the most common natural zeolites (An 1978; Colella 1999; Inglezakis and Zorpas 2012; Joshi and Mohan Rao 1983; Mohammadzadeh Kakhki et al. 2023; Pabalan and Bertetti 2001)

**Zeolite**	**Si/Al**	**CEC**^**a**^ **(meq/g)**	**Exchange selectivity**	**Site of exchange**
Clinoptilolite	4.0–5.3	2.2	Cs^+^ > K^+^ > Sr^2+^> Ba^2+^ > Ca^2+^ > Na^+^ > Li^+^ Pb^2+^ > Ag^+^ > Cd^2+^ > Zn^2+^ > Ca^2+^ > Na^+^	Channels
Chabazite	1.4–2.8	3.9	Ti^4+^ >Cs^+^ >K^+^ >Ag^+^ > Rb^+^ > NH_4_^+^ > Pb^2+^ Na^+^ = Ba^2+^ > Sr^2+^ > Ca^2+^ > Li^+^	Cavities
Phillipsite	1.3–2.9	4.5	Ba^2+^ > Rb^+^ > Cs^+^ > K^+^ > Na^+^ > Li^+^	Channels
Mordenite	4.4–5.5	2.2	Cs^+^ > K^+^ > NH_4_^+^ > Na^+^ > Ba^2+^ > Li^+^ NH^4+^ > Na^+^ > Mn^2+^ > Cu^2+^ > Zn^2+^ > Ni^2+^	Channels
Heulandite	3.0–3.2	3.2	Cs^+^ > K^+^ > Na^+^ > Li^+^ Ba^2+^ > Sr^2+^ > Mg^2+^	Channels

^a^Cation-exchange capacity (CEC) of natural zeolites based on the number of equivalents of exchangeable cations or the number of moles of Al^3+^ in the chemical formula

Based on the value reported in Table 3, it is clear that clinoptilolite and mordenite have higher si/al ratios compared to chabazite, heulandite, and phillipsite, which means lower content of aluminum; thus, negative charges in the structure, so low basicity. Moreover, this ratio is also important for the stability. In fact, zeolites with higher silica content, such as clinoptilolite, are stable under acidic conditions (Mohammadzadeh Kakhki et al. 2023). Furthermore, the Si/Al ratio influences hydrophobicity/hydrophilicity properties. Precisely, zeolites with lower Si/Al ratios interact more strongly and with a greater amount of H_2_O molecules, since the presence of a higher number of negatively charged oxygens in the framework and compensating cations will lead to more regions with charge separation. On the contrary, a high Si/Al ratio, as in the case of clinoptilolite, brings more hydrophobic zeolites (Anderson and Klinowski 1986; Bacariza et al. 2019). This behavior can be beneficial for those applications where the competitive effect of water with other molecules should be avoided, i.e., CO_2_ reduction to methane. It is important to mention that Si/Al also influences the so-called cation-exchange capacity. This parameter is directly proportional to the number of Al atoms present in the tetrahedral structure. In fact, when the Si/Al ratio is higher than 4, zeolite results in a relatively low ion exchange capacity (Álvarez et al. 2021). However, it should be emphasized that the exchange capacity of a zeolite towards a given cation depends on both the concentration of the ions in the solution and the affinity between the cation and zeolite, also known as selectivity. It is also important to remember that the exchange process can be limited by steric factors. Therefore, the relationship between the size of zeolite channels and the size of exchangeable cations plays an important role (Rodríguez-Iznaga et al. 2022). The CEC will be discussed in more detail in the following section. Moreover, it is noteworthy to mention that the Si/Al ratio also affects the nature of exchange cations, depending on the atomic radius. Precisely, from the literature (Rodríguez-Iznaga et al. 2022; Roque-Malherbe 2001), it is known that the zeolites which possess a high Si/Al ratio are more likely characterized by the presence of cations with larger ionic radii (i.e., alkali metals). For example, this is the reason for which clinoptilolite is richer in alkali metals compared to heulandite. It should be noted that a plausible explanation for this has still not been found. Nevertheless, some researchers have advanced a thermodynamic interpretation strictly correlated to the ion exchange which involves the heat of hydration of the various species involved. Specifically, an inverse correlation between the Si/Al ratio and heat of hydration was proposed. Taking into account this, cations with low hydration heats (i.e., alkali metals) are selectively exchanged by natural zeolites with high Si/Al ratio, like clinoptilolite.

#### Ion exchange

Ion exchange is a process thanks to which zeolites are able to exchange ions and modify their properties, i.e., their selectivity toward specific compounds. As previously described, the framework of zeolites is composed by micropores with an average size of less than 20 Å, which usually host water molecules or metal cations. However, these cations do not belong to the structure of the zeolite itself and, consequently, they can be substituted without affecting the framework’s stability (Rodríguez-Iznaga et al. 2022). Additionally, the presence of water molecules could affect the position of interchangeable cations. In fact, if cations are strongly hydrated or solvated, they do not tend to share water molecules with other sites, thus affecting the ion exchange (Higgins et al. 2002). When zeolites are in contact with electrolyte solutions, they are able to exchange their extra-framework cations with the ones present in the aqueous phase. In this process, two different types of ions are involved (Roque-Malherbe 2001): (i) extra-framework ions for charge balance and (ii) ions dissolved in the electrolyte solution. Moreover, in this process, three phases with a different kinetic can be identified. Precisely, the first step is characterized by the interdiffusion of cations in the thin adherent liquid layer; in the intermediate step, both interdiffusion in thin liquid layer and crystalline interdiffusion occur; finally, the last phase involves interdiffusion of cations in the zeolite crystals (Roque-Malherbe 2001). It is important to remember that the bond between exchangeable cations and the anionic framework is weak and this fact explains why cations can be theoretically removed by rinsing with a concentrated solution of another cation. However, the ion exchange process is strictly reliant on several factors, including the framework topology, the polarizability (ion size and shape), the charge density of the anionic frame, and the ionic charge as well as the concentration of the electrolyte solution (Barrer 1978). Furthermore, regarding natural zeolites, Gottardi et al. (Gottardi and Galli 2012) found that the number of tetrahedral Al^3+^ must be equal to the sum of positive charges related to exchangeable cations. Therefore, the cation exchange capacity (named CEC) of a natural zeolite, defined as the number of milliequivalents of cations exchangeable per gram of zeolitic material, is strictly dependent on the charge density of the anionic structure (thus, the degree of substitution of Si^4+^ with Al^3+^ in framework). In conclusion, the greater the aluminum substitution, the greater amount of cations is needed to guarantee electroneutrality; thus, the greater is CEC (Bish and Ming 2018). There is also a numerical relation which correlates the total exchange capacity (expressed as *m*_eq_/g) and the number of Al atoms per framework unit cell (Roque-Malherbe 2001). In clinoptilolite, for example, this parameter ranges from 2.19 to 3.11 m_eq_/g.

Finally, it should be highlighted that the evaluation of CEC related to a natural zeolite, such as clinoptilolite, is challenging due to the impurities and other phases depending on zeolites’ origin or on the process of purification they attended (Rodríguez-Iznaga et al. 2022). In any case, the relation between the nature of exchangeable cations and the location of exchange sites is considered a key property. In fact, natural zeolites with high Si/Al ratio, i.e., clinoptilolite, chabazite, and mordenite revealed high selectivity towards Cs^+^, Rb^+^, Na^+^, K^+^, and NH_4_^+^ since zeolites with high Si/Al ratio developed within their structure a minor quantity of heat respect to the one evolved in the solution. Consequently, cations with low hydration heats (as in the above-mentioned cases) are selectively exchanged by zeolites (Roque-Malherbe 2001).

#### Zeodratation: the effect of water adsorption and desorption

**Fig. 11:**
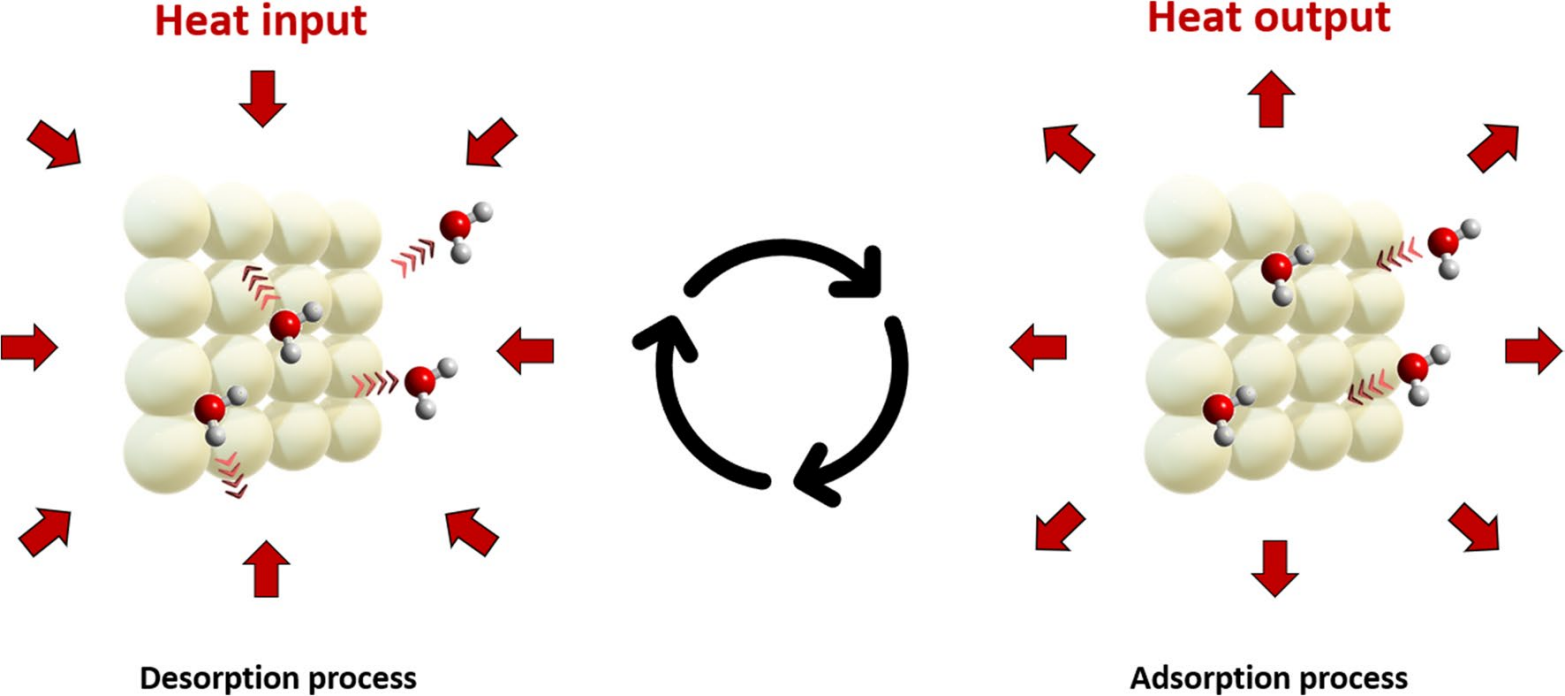
Schematization of the zeodratation phenomena occurring in a zeolite. Adapted from (Banaei and Zanj 2021)

Zeolites are known to be perfect sorbents for water, which is directly connected with framework aluminum (Gackowski and Paczwa 2021). It is well known that the void present between TO_4_ tetrahedra in zeolite’s structure can host up to 30 wt% of water. Since zeolites are hygroscopic, they are able to adsorb water molecules in the adjacent free spaces in their framework, blocking their mobility. As a result, the kinetic energy of water molecules (due to their Brownian motion) is converted into useful heat available for different applications. This is a reversible process since, when temperature increases, water can be expelled from zeolite’s framework as water vapor, thus generating condensation heat that can be exploited in a wide variety of applications. In other words, the ability to adsorb and absorb water cyclically represents the main advantage of zeolites as innovative materials for thermal energy storage (see Fig. 11). Additionally, it should be emphasized that both synthetic (13X) and natural (Clinoptilolite and Chabazite) zeolites have been investigated for this application (Banaei and Zanj 2021; Van Reeuwijk 1974).

It is noteworthy to highlight that when ion exchange is performed, the water adsorption capacity of clinoptilolite may be modified due to the presence of cations with different charges and/or ionic radii in the framework (Castaldi et al. 2005). In fact, the location of water molecules can vary according to the shape and the cavities of channels, but also due to the number of cations and the environmental conditions in which the zeolite is placed (M. Mohamed 2001). As an example, Castaldi et al. (2005) exploited both thermal analysis and Fourier-transform (FT) infrared (IR) spectroscopy to analyze the activation energy of dehydration and thermal stability of clinoptilolite. They also investigated the effect of extra-framework cations (Na^+^, K^+^, NH_4_^+^, Pb^2+^, Cd^2+^, and Zn^2+^) introduced by cation exchange in clinoptilolite’s structure. As a result, the thermo-analytical data proved the existence of three “dehydration steps” (depending on the type of water lost), occurring at three different temperatures. Precisely, weakly bound water was lost in the range between 60 and 150 °C whereas at 150–250 °C it was possible to observe the loss of water located in the cavities and bound to extra-framework cations. They also observed that the amount of water lost in this region turned out to be higher for clinoptilolite exchanged with bivalent cations and this happened because of the grater interaction between these cations and water, which resulted also in higher thermal stability. Finally, between 450 °C and 500 °C, structural water is lost and then the destruction of zeolite’s framework occurs. In a study led by Alver et al. (2010), a Turkish clinoptilolite exchanged both with monovalent and with bivalent cations was investigated for the water loss. Moreover, a Turkish clinoptilolite exchanged both with monovalent and with bivalent cations was investigated for the water loss. The authors found that decreased inversely proportional to the size of the extra-frame cations. Moreover, regarding thermal stability, it improved with the increase of the alkaline behavior of the zeolite that was dependent on the electro-positivity of exchanged cations.

## Environmental applications

Natural zeolites, and in particular clinoptilolite, have been extensively exploited in the field of environmental remediation and restoration, above all for their ion-exchange properties. In particular, clinoptilolite, in its both natural and modified form, has proven to be extremely attractive in the separation, binding, and chemical stabilization of hazardous inorganic, organic, and radioactive species in soils and aqueous systems (Misaelides 2011). Not only that: over the years, several studies have reported the effectiveness of removing pollutants from gas streams, such as carbon dioxide, methane, and ammonia, in some cases reporting performance comparable to that of synthetic zeolites (Roque-Malherbe 2001). In addition to its ion exchange properties, which make clinoptilolite suitable for applications in environmental remediation, wastewater treatment, water softening, groundwater remediation, and so on, another extremely important property is its affinity for water, which makes it suitable for removing moisture and impurities from gases, thus acting as a desiccant. This is particularly interesting in air conditioning systems, natural gas drying, and protecting sensitive products from moisture damage (Bish and Ming 2018; Nakhli et al. 2017; Rodríguez-Iznaga et al. 2022; Saravanan et al. 2021). Therefore, the use of clinoptilolite has revealed numerous advantages, including its low cost, high availability in nature, good mechanical and thermal properties, and above all high adsorption capacity, as described in the previous sections. In the following paragraphs, we have tried to go into more detail about the various fields of application, dividing them into three main categories, specifically, (i) pollution control (both in gaseous and aqueous streams); (ii) catalysis; and (iii) agriculture and livestock breeding, trying to provide results that can be considered starting points for continuing to explore the potential of clinoptilolite.

### Pollution control in gaseous stream

**Fig. 12:**
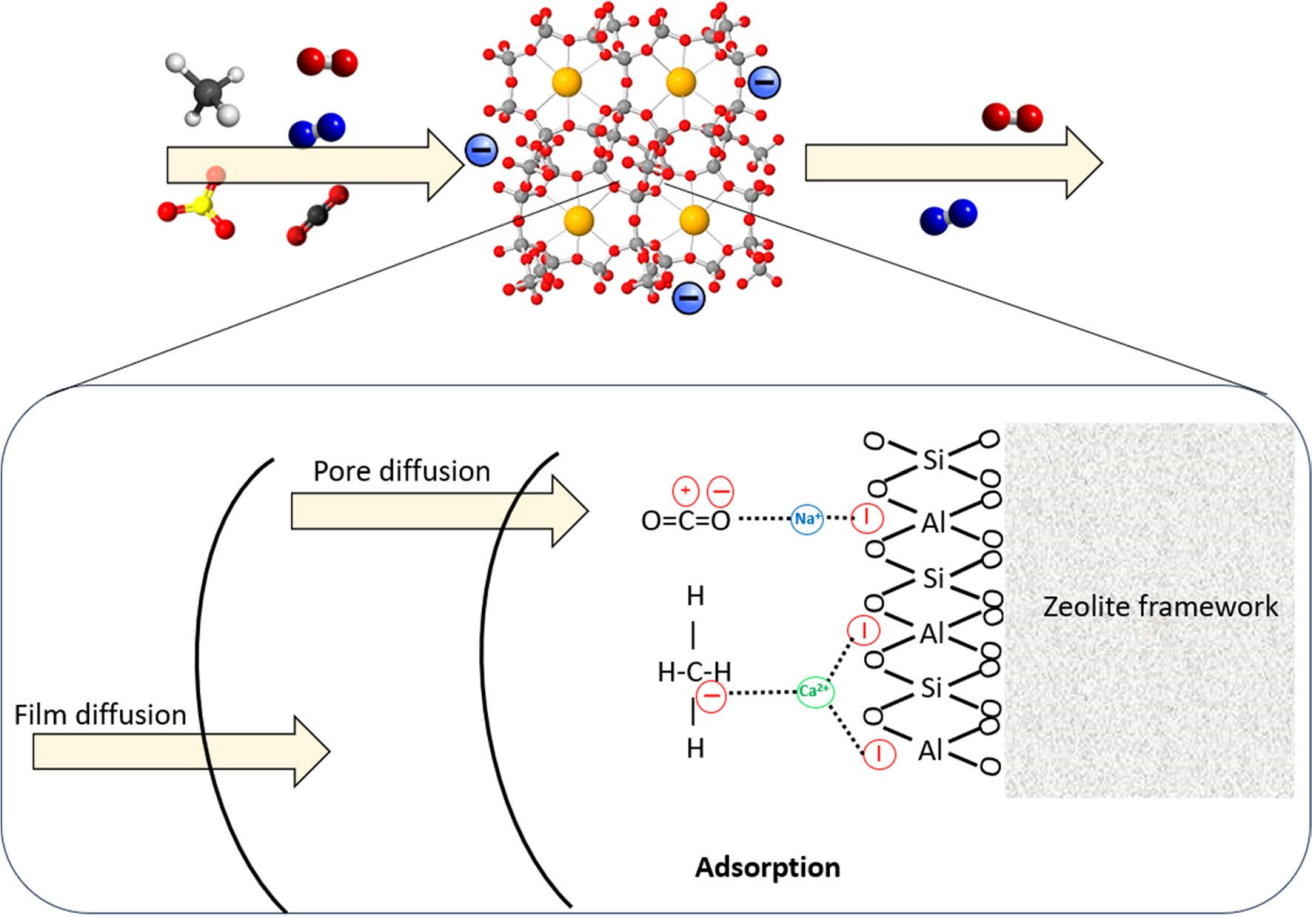
Schematic representation of pollutants adsorption for the purification of gaseous stream

One of the major causes of climate change is the excessive presence in the atmosphere of dangerous greenhouse gases, mainly carbon dioxide (CO_2_), which persist for a hundred years after its emission. To control human activities and decrease CO_2_ emissions, many protocols have been implemented in different nations (Attila 2021; Breidenich et al. 1998). Despite all these efforts, this purpose has been revealed to be particularly challenging because of humanity’s reliance on fossil fuels, whose combustion leads to CO_2_ release, in particular for energy production (Change 2014; de Gennaro et al. 2022). In the last decades, natural zeolite clinoptilolite has been subjected to intense research for its application as sustainable and low-cost sorbent for the purification of gaseous streams from different pollutants. A schematic representation of the adsorption process is reported in Fig. 12.

In addition, due to its properties, mainly excellent thermal stability, acid resistance, and high CO_2_ selective adsorption, this natural zeolite can be exploited in carbon capture, utilization, and sequestration (named CCUS) processes (Cavallo et al. 2023; Davarpanah et al. 2020; Diógenes et al. 2022; Montes Luna et al. 2018).

**Fig. 13:**
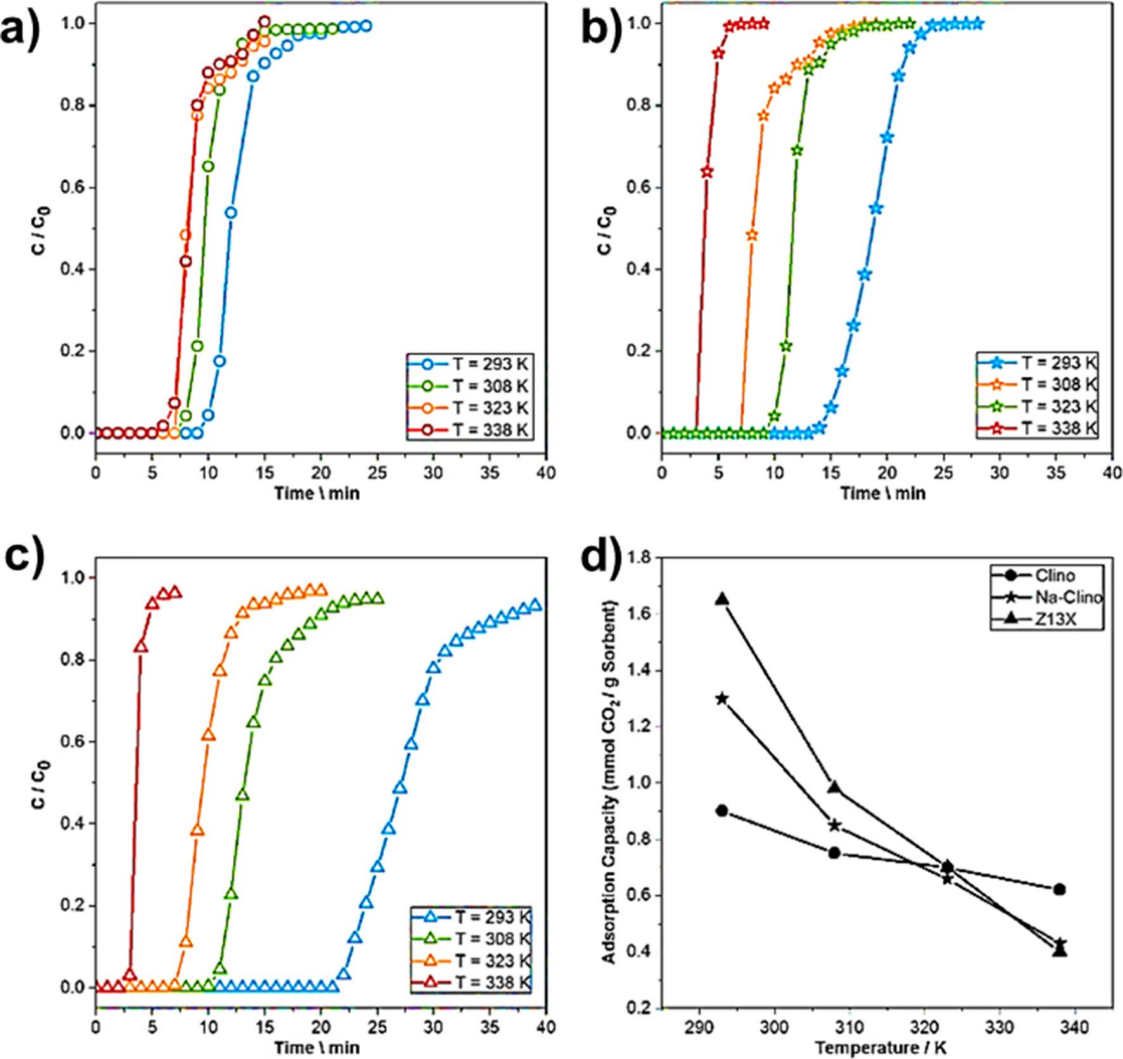
Adsorption breakthrough curves measured **a** for clinoptilolite; **b** Na-clinoptilolite; **c** Z13X at 293 K, 308 K, 323 K and 338 K. **d** Comparison of the CO_2_ adsorption capacities for the three samples as a function of the temperature, as derived from the breakthrough curves. Adapted from (Davarpanah et al. 2020) with the permission of Elsevier

It is known that CO_2_ capture can be achieved through traditional and well-known processes like cryogenic distillation, absorption by amines, and permeation membranes. In particular, amine-based regenerative adsorption processes have been exploited for several years, not only for the adsorption of carbon dioxide, but also for the separation of H_2_S from natural gas and other refinery processes. However, the high cost of this technique coupled with its high energy consumption led to the search for alternative strategies, among which the adsorption process truly represents a valid candidate. Among the advantages, it is useful to mention the reversibility of the process, the high selectivity, and the low energy demand for regeneration of the adsorbent material compared to other techniques. Different adsorbent materials are suitable for this purpose, i.e., activated carbon, alumina, silica gel, metal organic frameworks (MOFs), and, above all, zeolites (Samanta et al. 2012). Regarding structural characteristics influencing the carbon dioxide uptake, basicity plays an important role. Indeed, clinoptilolite naturally presents a series of cations whose alkaline properties increase the electron density of the framework oxygen, thereby allowing the capture of acidic molecules (Naccache 1987). For example, Siriwardane et al. (2003) tested three different natural zeolites, including clinoptilolite, as possible sorbents in the pressure swing adsorption (PSA) process, known to be clean and economically attractive technology and efficient processes both for CO_2_ capture and for gas streams purification (Sircar 2002). They performed test to separate CO_2_ from flue gas at room temperature and at a pressure of 2×10^6^ Pa and discovered that clinoptilolite with a higher amount of sodium was more efficient in the CO_2_ adsorption process, since it had an influence on both the local electric field and the polarization of the adsorbed molecules on the zeolites (Hernández-Huesca et al. 1999). Moreover, CO_2_ adsorption capacity strictly depends on Si/Al ratio and pore size (Bonenfant et al. 2008; Hernández-Huesca et al. 1999). Precisely, clinoptilolite is only able to adsorb molecules with a maximum kinetic diameter of 3.5/3.8 Å thus demonstrating that CO_2_ (which present a kinetic diameter of 3.3Å) adsorption is favored (Breck 1973). Finally, it should be mentioned that carbon dioxide uptake is favored by low temperatures and high partial pressures and it is strictly obstructed by the presence of water which lowers the strength of the electric field as well as induces the formation of bicarbonates (Bonenfant et al. 2008). Therefore, the operative conditions have an important role in this process. In another study, Diògenes et al. (2022) exploited both fixed bed experiments and molecular simulations to test clinoptilolite adsorption performance towards a mixture of CO_2_, N_2_, CH_4_, and O_2_ and through column dynamics they confirmed the high selectivity of this natural zeolite towards carbon dioxide. To enhance the sorption capacity and selectivity toward different molecules and in particular with respect to CO_2_, some structure modifications could be introduced by improving the adsorbent pore size or by exchanging the cations, but also increasing the active surface area as well as pore volume, in order to obtain a stronger polarization effect and higher contact area and then stronger polarization. This is feasible thanks to the presence of Van Der Waals forces and electrostatic interactions between CO_2_ and clinoptilolite (De Souza et al. 2018; Dosa et al. 2021; Kurama et al. 2002). For instance, Davarpanah et al. (2020) demonstrated that the adsorption capacity of clinoptilolite towards carbon dioxide at 293K in both dynamic and equilibrium conditions can be enhanced thanks to ion-exchange modification with Na^+^ (see Fig. 13). Moreover, despite synthetic zeolite 13X exhibited the best performance at 293 K, bare clinoptilolite showed the highest CO_2_ uptake of 0.6 mmol CO_2_/g at 338K, highlighting its potential application.

In another study carried out by Mortazavi et al. (2021), clinoptilolite was doped by using different procedures including an ionic exchange with Li^+^, Mg^2+^, and Ca^2+^ ions, modification with monoethanolamine (MEA), triethanolamine (TEA), and hexyl amine and by using ionic liquid modification with PF_6_^−^, NO_3_^−^, Cl^−^, and Br^−^. All the samples investigated exhibited a better performance probably caused by a stronger interaction between CO_2_ molecules and the modified substrates. On the other hand, Wahono et al. (2020) demonstrated that it was possible to obtain a competitive alternative to synthetic zeolites for CO_2_ removal with an Indonesian mordenite-clinoptilolite zeolite, dealuminated through 12M HCl and then calcinated at 400°C. Indeed, these physico-chemical treatments increased both of the specific surface areas (from 26 m^2^/g to 179 m^2^/g) and pore size, thereby enhancing CO_2_ uptake.

Clinoptilolite can also be used for the separation of other gases, besides CO_2_. For example, in a study carried out by Alonso-Vicario et al. (2010), this material was used both for the removal of H_2_S and upgrading the biogas and then compared with a synthetic zeolite (13X). The results demonstrated that clinoptilolite was able to clean and upgrade the biogas at the same time under the operative conditions of 7 bar and 25 °C exhibiting an adsorption capacity of 1.39 mg of H_2_S/g and 173 mg CO_2_/g which was higher than those of 13X. In another work, Ciahotný et al. (2002) studied a Slovakian clinoptilolite for the adsorption of ammonia from waste air and its performance was compared with the same sample modified with acids, mainly H_2_SO_4_ and H_3_PO_4_. The pristine sample showed a considerable adsorption capacity towards ammonia (10.8 mg NH_3_/g sorbent at 20 °C); however, the material treated with acids exhibited a higher quantity of ammonia adsorbed at the same temperature (21.6 and 20.0 mg NH_3_/g sorbent, for H_2_SO_4_ and H_3_PO_4_ respectively). This happened because the combination of several mechanisms contributed to the enhancement of the sample’s adsorption capacity. Furthermore, Meimand et al. (2019) tested clinoptilolite both in its natural form and as support for the dispersion of iron oxide nanoparticles with the aim of adsorbing sulfur dioxide gas (SO_2_) which negatively affects both humans and the environment. The results showed promising results since clinoptilolite showed an adsorption efficiency of 66.7% in its natural form and of 80.3% when utilized as support, corresponding to 43.8% and 31.3% of removal from the stream at room temperature.

### Pollution control in wastewater

Water is one of the most important resources on the Earth; thus, its purification processes are crucial to ensure drinking water for people and that it is suitable for agricultural purposes. Due to industrial growth, wastewater pollution increased over the years, therefore treatment with cost-effective technologies and sustainable materials to preserve public health and reduce levels of environmental degradation (Kumar 2013; Nafea et al. 2024; Saravanan et al. 2021).

**Fig. 14:**
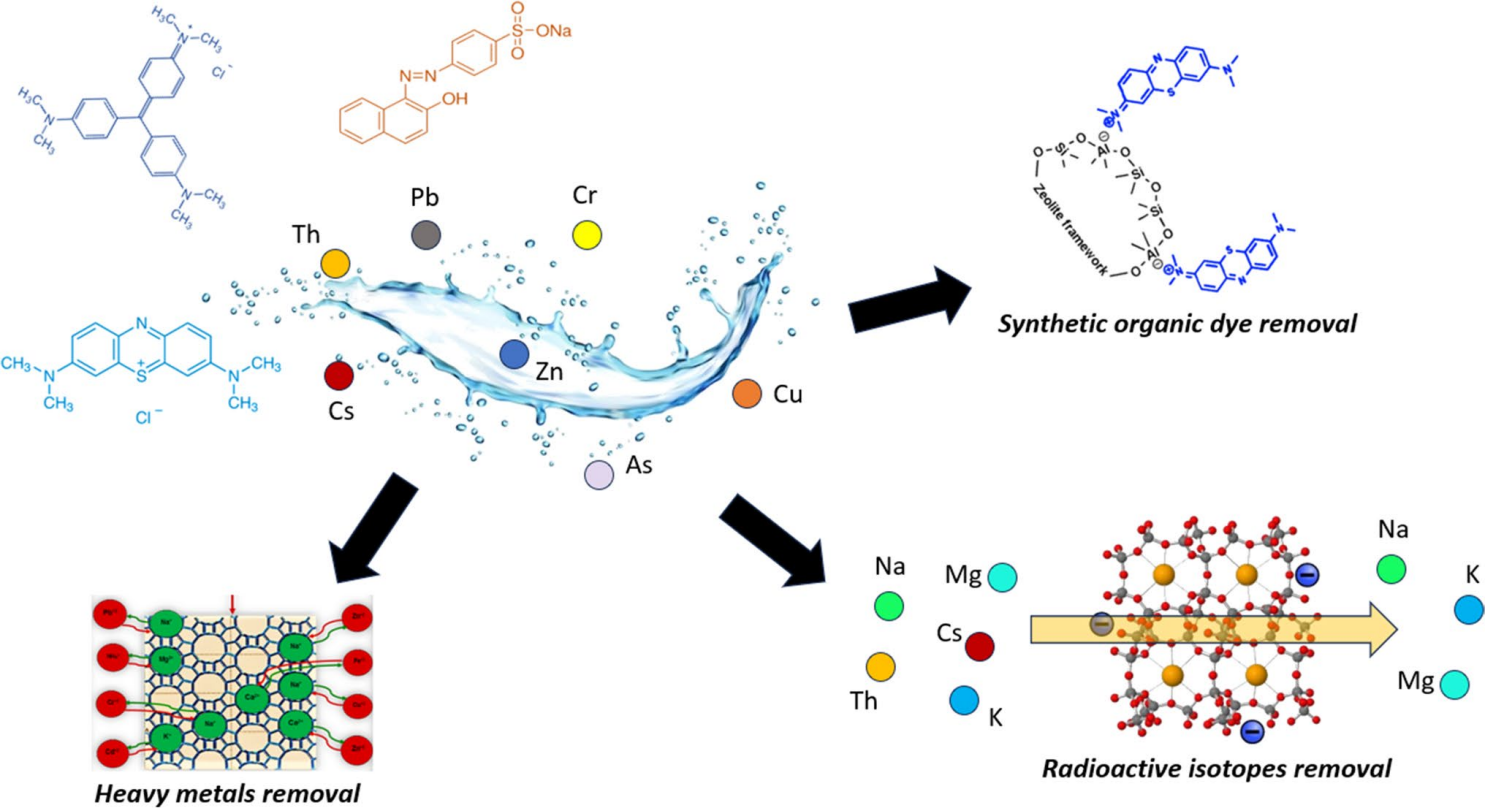
Different uses of clinoptilolite for the purification of wastewater streams through adsorption process

Different types of pollutants could be present in wastewater, i.e., synthetic organic dyes, heavy metals, pharmaceuticals, etc. (see Fig. 14). They derive from the discharge of various industries like the battery industry, petroleum refining, pesticides, and textile industry. In this context, heavy metals represent an important issue since it could lead to bioaccumulation and hazardous acute and long-term effects on human health and the environment (Ahmed et al. 2023; Kumari and Bhattacharya 2023). For example, Pb can damage the kidneys, the nervous system, and the brain whereas Cd can cause the illness of hepatic injury, renal dysfunction, and lung damage (Liu et al. 2023; Zheng et al. 2023). To protect human health and the ecosystem, the World Health Organization (WHO) (WHO 2011) has published guidelines for water quality with very rigorous limits concerning the presence of heavy metals in drinking water. Another important class of pollutants are synthetic organic dyes, i.e., Methylene Blue, Crystal Violet, pigments, and so on. They are classified as micropollutants due to their low concentration (ng/l to μg/l) and exhibit toxicity and non-biodegradability (Tkaczyk et al. 2020; Yusuf 2019). Therefore, it is essential to develop solutions for the removal of these pollutants and among them, adsorption has been revealed to be the most cost-effective and highly efficient strategy. The most important role in this process is certainly played by the proper absorbent, whose effectiveness depends on the specific surface area, porosity, functional groups, and so on. The most common adsorbent is activated carbon since it exhibits high efficiency in the removal (Bouchelkia et al. 2023; Fito et al. 2023; B. A. Mohamed et al. 2023; Obayomi et al. 2023; Raninga et al. 2023; Valério Filho et al. 2023). However, due to its high cost which depends on chemical activators, high regeneration temperatures, etc. (Dosa et al. 2021; Zhao et al. 2023), Scientific Community became more interested in other sustainable alternatives for wastewater treatments. In this context, clinoptilolite represents an ideal candidate for the removal of pollutants from waste fluids thanks to its excellent selectivity for a variety of heavy metals (Li et al. 2019), including radioactive isotopes like Cs^+^ and Sr^2+^ (Lora 2018), and the presence of electrostatic forces in the cavities which allow the interaction with several compounds. Some examples of pollutants removed by clinoptilolite for wastewater purification are listed in Table 4.

**Table 4:** Examples of molecules removed by using clinoptilolite and other applications in wastewater treatment

**Application**	**Material**	**Experimental**	**Performance**	**Ref.**
Bisphenol A removal	Clinoptilolite (From Romania)	*BPA concentration:* 1–300 mg/L *Adsorbent dose:* 1g/L *Temperature:* 20 °C	*Max. adsorption capacity:* 12 mg/g at pH=4.11 50 mg/g at pH=8.12 *Best model fitted:* Langmuir model	Dura et al. (2023)
Chromium removal	Clinoptilolite	Fixed bed and batch studies carried out *Cr(VI) concentration* 10 mg/L *pH*: 5.2 *Column height:* 50cm *Column diameter:* 1cm *Flowrate:* 5ml/min	*Adsorption capacity with fixed bed studies*: Bare clinoptilolite: 2.2 mg/g NaCl-treated clinoptilolite: 4.5 mg/g *Adsorption capacity with batch studies:* Bare clinoptilolite: 1.8 mg/g NaCl-treated clinoptilolite: 3.2 mg/g	Kurniawan et al. (2023)
Support for bacterial community formation	Clinoptilolite (From Romania)	*Adsorbent dose:* 100 g/L *Wastewater composition:* Ammonia, nitrates and nitrite, dye, metals *Experiment duration* 30 days *Particle size range*: (1–3 mm), pH=8.2 <10μm, pH=8.4	*NH*_*4*_^*+*^ *removal efficiency* >98%	Senila et al. (2022)
Ammonia removal	Clinoptilolite (From China)	*Volume solution*: 100 ml *Ammonia concentration:* 50 mg/L *Adsorbent dose*: 0.5 g *Temperature:* 27 °C	*Ammonia removal efficiency* > 82.97% after 180 min	Adam et al. (2023)
Ammonia adsorption and recovery	Clinoptilolite (From USA)	Batch experiment *Ammonia concentration*: 400 ppm pH 8	*Ammonia adsorption capacity* 2.97–4.6 mg/g *Desorption efficiency* above 78% for 4 cycles *Best model fitted:* Pseudo-second-order model	Kannan and Parameswaran (2021)
Heavy metal removal	Clinoptilolite (From Bulgaria)	*PART 1* *Heavy metals:* Cu(II), Zn(II), Mn(II), and Pb(II) *Initial concentrations* 5, 25, 50, 200, and 400 mg/l *Adsorbent dose:* 50 g/L *Initial pH* 3.5 PART 2 *Heavy metals:* Co(II), Ni(II) *Initial concentrations* 350–650 μg/l *Adsorbent dose:* 2.5 g/L *Initial pH* 6	*Maximum adsorption capacity*: Pb 30 mg/g Cu and Mn 4.5 mg/g Zn, Ni, and Co 3.5 mg/g *Removal efficiency* Cu(II) 97.84% Zn(II) 94% Mn(II) 89.6% Pb(II) 97.6% Co(II) 98.8% Ni(II) 88.88% *Best model fitted*: Langmuir-Freundlich isotherms	Zendelska et al. (2018)
Methylene Blue removal	Fe_2_O_3_ NPs/clinoptilolite (From Iran)	*Initial concentration* 25–200 mg/L *Adsorbent dose:* 0.1–1 g pH effect investigated	*Removal efficiency* 26.86% at pH 3 48% at pH 9 *Adsorption capacity* 96.4–98.6% (independent from pH) *Dye removal* >98% at an optimal contact time of 45 min	Badeenezhad et al. (2019)
Complex textile effluents removal	Clinoptilolite doped hydrogels (From Turkey)	*pH* 5.0–9.0 *Temperature* 25–40 °C *Reaction time* 60–120 min *Adsorbent dose* 0.5–2.0 g/50 mL *Zeolite percentage* 1–6%	*Adsorption capacity* 559.47 mg/g *Adsorption isotherms* Langmuir model *Optimum color removal* 86% *Best model fitted* Pseudo second order model	Doğaroğlu et al. (2023)

#### Removal of synthetic organic dyes

**Fig. 15:**
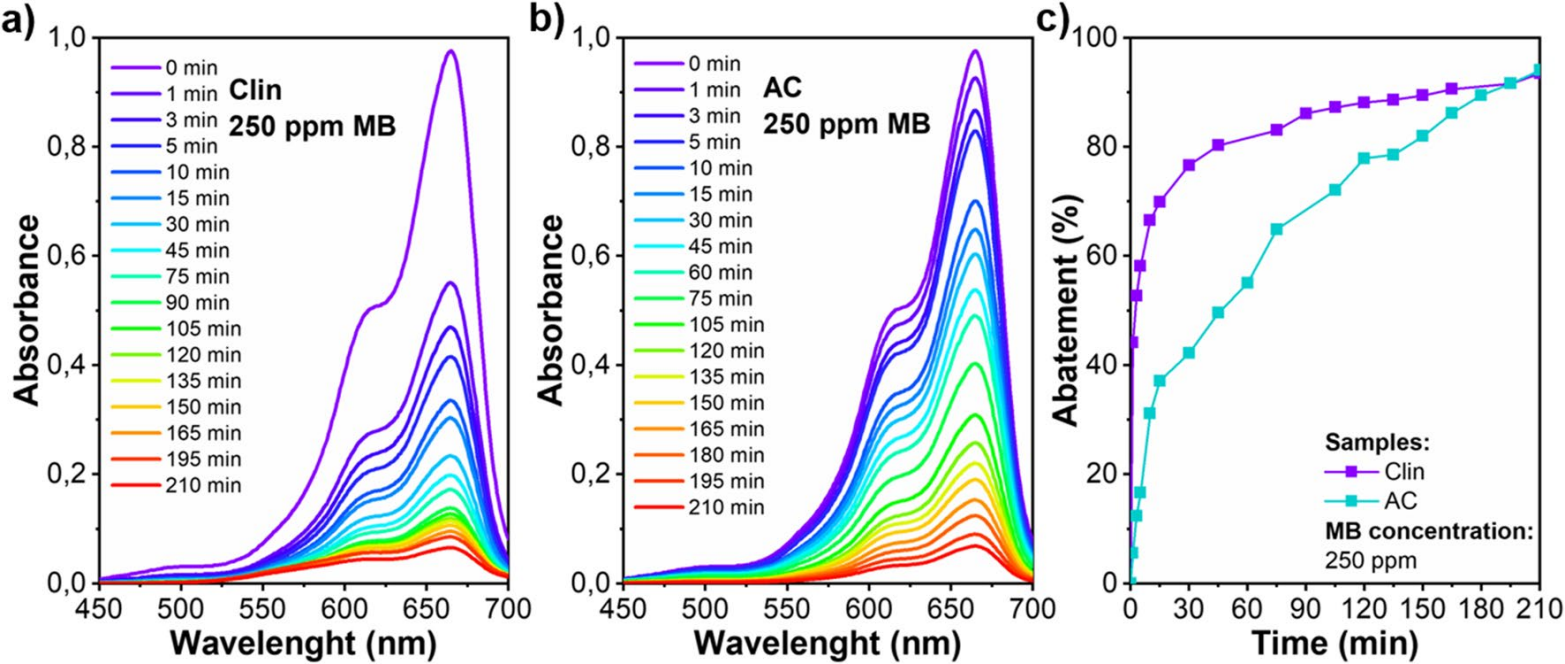
Comparison of clinoptilolite (Clin) and activated carbon (AC) for the removal of Methylene Blue (MB). Adapted from (Dosa et al. 2022)

In literature, it is possible to find different works concerning the use of natural clinoptilolite for the removal of dyes (Amin et al. 2023; Dosa et al. 2018). For example, a specific use of the clinoptilolite could be the removal of pollutants, i.e., synthetic organic dyes, from wastewater derived from textile industries. In one of our works (Dosa et al. 2022), we investigated the use of Greek clinoptilolite (labeled as Clin) for the adsorption of Methylene Blue (MB), used as a target molecule from textile wastewater. Precisely, in the first part of the work, the effect of different concentrations of MB on the performance of the clinoptilolite was studied, observing that it was able to capture 100%, 99%, and 93% respectively with an initial concentration of 100, 200, and 250 ppm of dye after 210 min and introducing an adsorbent load of 10 g/L. In the second part, we compared the performance of Clin with that of activated carbon (named AC) with the aim of evaluating the use of a natural zeolite as a possible sustainable and cost-effective alternative to the use of AC. In this case, adsorption tests were performed with the highest concentration of pollutant (250 ppm) and a concentration of adsorbent equal to 5 g/L. As reported in Fig. 15, it was observed that the removal with clinoptilolite is much faster, reaching almost 70% removal in less than 30 min and 96% in 210 min, by following a pseudo-second-order model, highlighting that a chemisorption process effectively describes the adsorption behavior of MB. Moreover, clinoptilolite and activated charcoal were studied with the copresence of Methylene Blue and heavy metals (10 ppm of both Zn^2+^ and Cd^2+^). Interestingly, both adsorbents achieved 100% MB abatement (*t* = 40 min), but less removal of heavy metals, highlighting the preferential adsorption of the dye on active sites.

In another study, Nannu Shankar et al. (2023) investigated the adsorptive capacity of Indian clinoptilolite towards Violet 5BN (V5BN). The effect of different parameters showed that up to 96% of the dye was adsorbed by clinoptilolite at the adsorbent dose of 1.5 g, temperature of 30 °C, pH 5, and reaction time of 90 min. Moreover, they proved the process is spontaneous and characterized by chemisorption at a temperature lower than 30 °C whereas physisorption becomes predominant as temperature increases.

When the molecule is complicated to remove or there is a co-presence of different molecules, it could be useful to modify the clinoptilolite. For example, Noori et al. (2022) exploited the use of novel magnetic adsorbents, mainly clinoptilolite/Fe_3_O_4_ and alginate/clinoptilolite/Fe_3_O_4_, for the removal of Crystal Violet even with the co-presence of Methylene Blue. The adsorption tests showed that both materials exhibited excellent affinity to Crystal Violet reaching a removal of 94.32% and 92.35%, respectively, in the single system and of 84.19% and 80.23%, respectively in the binary system.

#### Removal of heavy metals

Clinoptilolite was also investigated for the removal of different heavy metals from the wastewater. Galletti et al. (2021) investigated its performance in a system comprising both zinc and cadmium ions, and observed that complete adsorption was reached when the metal concentration in the solutions was equal to 10 mg/L. Increasing the metal amount progressively to 50, 100, and 200 mg/L, adsorption removal decreased, mostly for Zn^2+^, down to 35% with 200 mg/L of metal in the starting solution, whereas Cd^2+^ ion removal was reduced much less, remaining above 50% even at high metal concentrations. The authors also investigated the different affinity in a system with the copresence of both heavy metals and saw a greater affinity of clinoptilolite toward Zn^2+^, highlighting competitive sorption phenomenon. Zanin et al. (2017) studied the use of natural Brazilian clinoptilolite as an adsorbent for the removal of heavy metals in wastewater from the graphic industry, by performing adsorption experiments for the removal of copper(II), chromium(III), and iron(III) and revealing adsorption up to 95.4% iron, 96.0% copper, and 85.1% chromium, at 25.0 °C and pH 4.0. They also found that zeolite selectivity followed the order Fe > Cr > Cu and the adsorption mechanism followed pseudo-first-order kinetic model for copper and chromium and pseudo-second-order for iron. Argun (2008) employed a Turkish Clinoptilolite for the removal of Ni(II) ions, reaching 93.6% of adsorption at pH 7 after 45 min of contact time for 25 mg/L initial concentration and a 15 g/L solid-to-liquid ratio.

Sometimes, the main obstacle concerning the use of clinoptilolite on a large scale is represented by the necessity of crushing it in a very fine powder before using it for water purification and, consequently, the difficulty of its reutilization. Consequently, some innovative technologies have been produced in these last decades to gain an optimization of the adsorption process, together with a better exploitation and reutilization of the materials. Among them, geopolymer-zeolite composites have recently started to gain particular interest for researchers. A geopolymer belongs to a class of amorphous inorganic polymers which are synthesized from an aluminosilicate source and alkaline solution. The most interesting thing about them is probably constituted by their zeolite-like structure as well as their excellent sorption properties towards heavy metals. Therefore, geopolymer-zeolite composite materials can be adopted as bulk-type adsorbents in industrial wastewater management, combining the micropores in zeolite’s framework with meso- and macro-pores of geopolymer, thus providing the hybrid structure with multiscale and interconnected pores. The obtained adsorbent could overcome not only all the difficulties related to reutilization of clinoptilolite powder, but also diffusion limitations problems linked to many industrial applications. The adsorptive properties of these hybrid adsorbents have been tested towards some target pollutants, i.e., Pb^2+^, Cd^2+^, Cu^2+^, Ni^2+^, and Cr^3+^ with promising results (Liu et al. 2023).

#### Removal of ammonia

Another application of clinoptilolite related to pollution control in wastewater concerns ammonia removal. Ammonia is a frequent contaminant in wastewater from industrial and municipal sources deriving from the development of the industrial sector, more precisely the field of coking, chemical fertilizers, coal gasification, petroleum refining, pharmaceuticals, and catalyst (Rožić et al. 2000). Since it constitutes a source of nitrogen, an excessive quantity may contribute to a considerable pollution burden, leading to fish toxicity as well as an acceleration of the process of corrosion of metals and other building materials, leading to expensive purification operations. In order to reduce operational costs, adsorption processes employing natural clinoptilolite have been investigated. However, the removal of ammonia is highly influenced by the presence of some cations, i.e., K^+^, Ca^2+^, Mg^2+^, and Na^+^, but also pH, temperature, and composition of clinoptilolite (Huang et al. 2010). Zabochnicka et al. (Zabochnicka and Malinska 2010) demonstrated that a Hungarian clinoptilolite exhibited the highest removal of ammonia from a solution containing 50 ppm, reaching 89.92–99.74% after 60 and 180 min of time exposure respectively. In another work, Adam et al. (2023) reported that more than 80% of the ammonia in the feed solution (containing 50 ppm) was effectively adsorbed by the natural zeolite clinoptilolite, highlighting high affinity with this molecule.

Finally, clinoptilolite can be also employed in multi-media biological aerated filters (MBAF) for synthetic water treatment (Ji et al. 2011). This technique possesses several advantages compared to conventional wastewater treatment solutions, i.e., activated-sludge, biological contact oxidation, and feedwater filtration. Its efficiency is mainly determined by the filtering media adopted and, in this context, clinoptilolite has demonstrated several advantages, including a rough surface, large adsorptive capacity, and low cost. In a work carried out by Zhang et al. (2007) the number of nitrobacteria grown on the clinoptilolite surface was 3.5 times higher than in sandy filter media, resulting in 70–90% removal of NH_3_-N and NO_3_-N, thus confirming the improved growth and reproduction of microorganisms, together with resistance to ammonia nitrogen shock, as the main advantages of natural zeolite.

#### Removal of radioactive isotopes

**Fig. 16:**
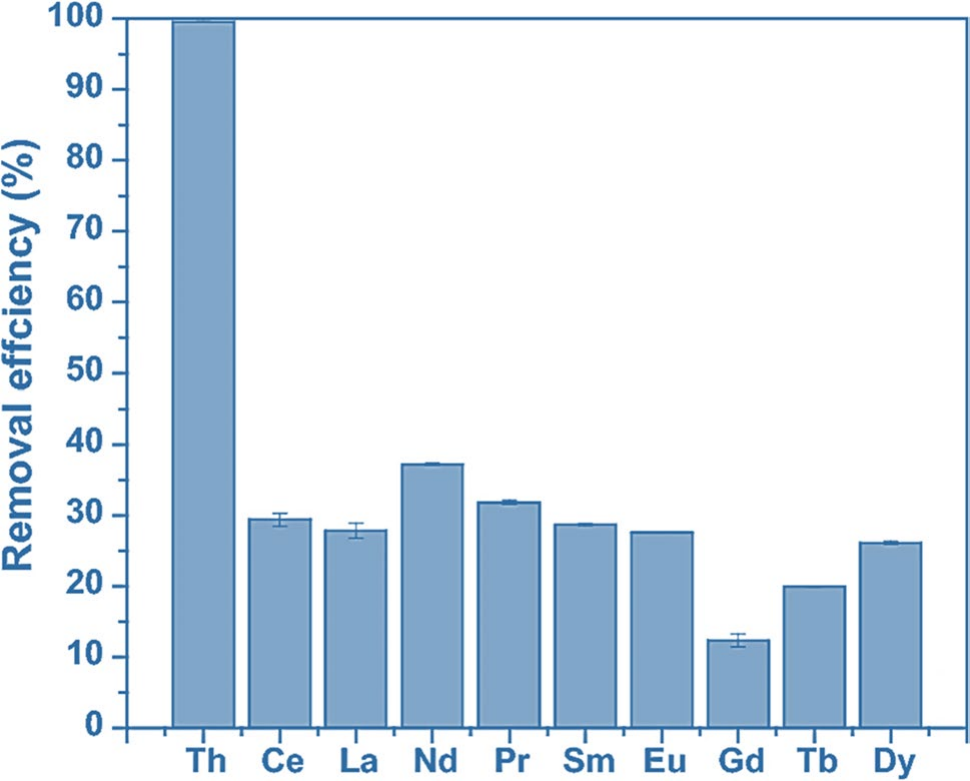
Clinoptilolite removal efficiency toward thorium and other metal ions from the leached solution. Adapted from (Alotaibi et al. 2023)

Recent scientific developments led to the investigation of the role of clinoptilolite as an adsorbent for radioactive compounds (Jiménez-Reyes et al. 2021). Actually, the fundamental mechanism is always the same involved for the adsorption of heavy metals that is ion exchange. However, the atomic radius plays an important role, as mentioned in previous paragraphs. Precisely, heavy metals (e.g., Pb, Cu, Fe, Mn) have small atomic radii (being on the right-hand side of the periodic table), whereas radioactive isotopes have larger radii. This influences the different selectivity and, in turn, is influenced by the Si/Al ratio. In fact, some researchers observed that natural zeolites with high Si/Al ratio, such as clinoptilolite, showed a not negligible selectivity towards large cations like Cs^+^, Rb^+^, Na^+^, and K^+^, compared to those that are smaller (like heavy metals). Alotaibi et al. (2023) investigated the performance of phosphate-modified clinoptilolite for the removal of thorium from aqueous solutions. Thorium can be released into the environment from natural and human activities. It is a radioactive element present on earth's crust and it is used in many industrial applications, including the nuclear fuel industry, since it could be converted to fissile material Uranium 233. The results of this study, shown in Fig. 16, revealed an adsorption efficiency of almost 99%, which is a very promising result. Other scientists tested this material for the adsorption of strontium in low-level liquid radioactive waste management, revealing a better affinity for strontium compared to other synthetic zeolites tested in the same context (Marinin and Brown 2000).

### Role of clinoptilolite as eco-friendly catalyst

**Fig. 17:**
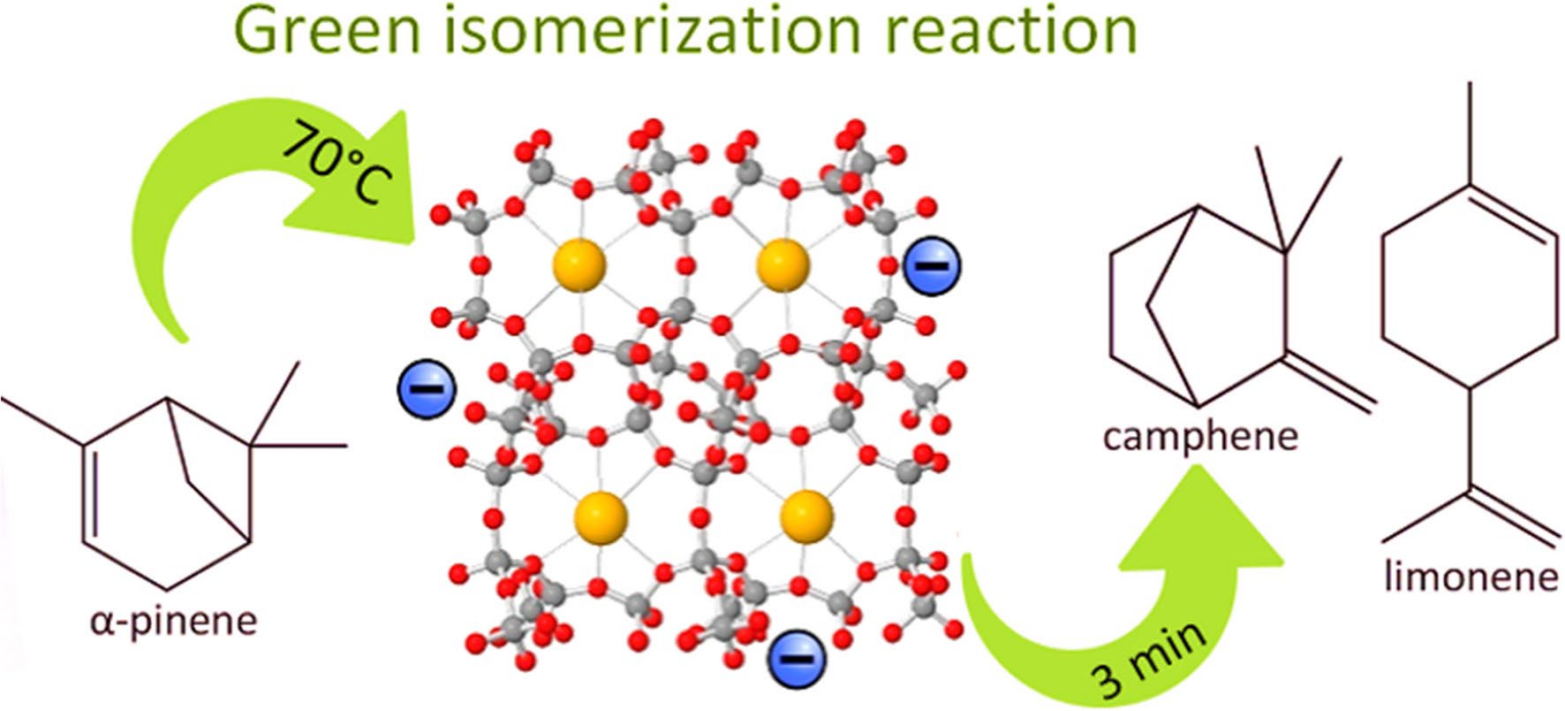
Solvent-free α-pinene isomerization process over acid-modified clinoptilolite. Adapted from (Miądlicki et al. 2021)

Natural zeolites, like clinoptilolite, were widely explored as potential candidates for catalysis, in order to minimize production costs and, at the same time, to overcome environmental challenges. In 2002, Kim et al. (2002) investigated the role of clinoptilolite in the degradation of polypropylene (PP). It is known that plastic waste can be thermally or catalytically degraded into gases to produce sources of energy and chemicals. Catalytic degradation is considered more convenient than the thermal one, since it requires lower temperatures and it allows to obtain precise products due to its selectivity which means a narrow distribution of hydrocarbons produced. In this context, the most used catalysts are solid acids (i.e., zeolites) and activated carbon. In this study, clinoptilolite, either in the proton-exchanged form or in the one treated by hydrochloric acid, resulted to be an efficient catalyst for the conversion of PP to gasoline-range chemicals at 400 °C, thanks to its acidic sites. In 2008, Royaee et al. (2008) employed this natural zeolite for catalyzing important petrochemical reactions, i.e., methanol dehydration to dimethylether (MTD), which is part of the methanol to olefin (MTO) and methanol to gasoline (MTG) processes. The main advantage in the use of clinoptilolite relies in a high selectivity, almost 99.7% obtained under industrial operative conditions of 16 bar. The material might be of high industrial interest because of the relatively lower activation energy (ca. 60 kJ/mol) compared to other zeolitic and non-zeolitic catalysts. Remaining in the petrochemical field, clinoptilolite-based catalysts are also used for increasing the octane number of gasoline, sometimes by coupling praseodymium oxide as a promotor of active site in the catalyst itself (Kusrini et al. 2018). Another example concerns the synthesis of eco-friendly heterogeneous CaO/clinoptilolite catalyst and, subsequently, its high-performance in biodiesel production from the transesterification reaction of methanol and waste cooking oil (Aghel et al. 2022). In another work, sulfuric acid–modified clinoptilolite was adopted as a green catalyst for solvent-free α-pinene isomerization process (see Fig. 17), to obtain high-value added chemicals (Miądlicki et al. 2021). The authors got 100% conversion after 3 min at 70 °C, with selectivities of 50% and 30% for camphene and limonene, which are considered industrially important products.

In another work carried out by Grzeszczak et al. (2023) in the same application field, the solvent-free oxidation of α-pinene over clinoptilolite reached 35 mol% of conversion and a selectivity of 29 mol%, 17 mol%, and 13 mol% toward α-pinene oxide, verbenol, and verbenone, respectively, under 100 °C, 0.05 wt% of catalyst, and 210 min of reaction time. These results are promising since the proposed method of oxidation is environmentally safe because it does not require the separation of products from the solvent. In addition, this method allows for managing the biomass in the form of turpentine, which is the main source of α-pinene.

Additionally, the potential of this natural zeolite was exploited for the synthesis of nanostructured catalysts, which were subsequently used in toluene abatement from waste gas stream at low temperatures. In this regard, CeO_2_ (30%)/clinoptilolite was synthesized through a treatment with HNO_3_ and co-precipitation methods, followed by the dispersion of 1% Pt over support. The synthesized catalyst was efficient for the oxidation of toluene, reaching 90% of abatement at 130°C (Amereh et al. 2018). In a similar work, Yosefi et al. (Yosefi et al. 2015a, 2015b) modified a clinoptilolite/CeO_2_ nanocatalyst with 5%, 10%, and 15% of Cu, respectively, by means of the sonochemical method, which is efficient in controlling pore size, morphology, and distribution of active phase on the support. The resulting catalyst was used for the abatement of toluene, obtaining the best removal over Cu (15%) clinoptilolite/CeO_2_, since it was able to abate 98% of pollutant even at high concentrations (3000 ppm). The same research team doped the same clinoptilolite/CeO_2_ nanocatalyst utilized in the previous study with different quantities of Mn (5%, 10%, and 15%) both via impregnation and the sonochemical method and then tested it for the removal of the toluene (Yosefi et al. 2015a, 2015b). The results showed that even with a low concentration of Mn, the catalyst was very active in the abatement of this pollutant, reaching almost complete removal at 350°C. Also, in this case, the realization of the abovementioned catalysts was performed to remove toluene from polluted air with not negligible conversion. Experimental results indicated Mn (15%) clinoptilolite/CeO_2_ as the best nano catalyst for this purpose. Similarly, the removal of xylene from waste gas stream at relatively low temperatures (250°C) was performed by Jamalzadeh et al. (2014) via catalytic oxidation over Pd/carbon-clinoptilolite-CeO_2_, obtaining 95% of abatement. Other reactions involving the use of clinoptilolite as a green catalyst are listed in Table 5. Recently, clinoptilolite has been used as a catalyst in other application field. In fact, in 2021, it was investigated for the isomerization of geraniol for medical applications (Fajdek-Bieda et al. 2021). In this study, different conditions were explored to obtain compounds like 6,11-dimethyl-2,6,10-dodecatrien-1-ol and thumbergol. These molecules are extremely valuable in medicine since they can be used in the treatment of cancer and may also have neuroprotective properties. The best results were obtained in 3 h at 140 °C, with the catalyst content of 12.5 wt%. At these conditions, the conversion of geraniol amounted to 98 mol%, and the selectivity of 6,11-dimethyl-2,6,10-dodecatrien-1-ol and thumbergol amounted to 14 and 47 mol%, respectively. The proposed method resulted very effective in obtaining these compounds with the advantage of the absence of a solvent in the reaction mixture.

**Table 5:** Examples of clinoptilolite-based catalyst

**Catalyst**	**Reaction**	**Ref.**
Fe-clinoptilolite	Acid Orange 7 degradation in presence of ascorbic acid and H_2_O_2_	Dosa et al. (2021)
Clinoptilolite	Etherification of glycerol	Hieu et al. (2022)
KOH/clinoptilolite	Carboxymethylation of hemicellulose	Khalilzadeh et al. (2019)
Clinoptilolite	Synthesis of 2-amino-4H-chromene derivatives	Baghbanian et al. (2013)
Hydrotalcite-modified clinoptilolite	Selective catalytic reduction of NO with ammonia (NH_3_-SCR)	Szymaszek-Wawryca et al. (2022)
TiO_2_/clinoptilolite	Photocatalytic degradation of xanthate	Shen et al. (2020)
HPA/clinoptilolite-Fe_3_O_4_	Synthesis of biodiesel from *Salvia mirzayanii* oil	Helmi et al. (2021)

### Agriculture and livestock breeding

**Fig. 18:**
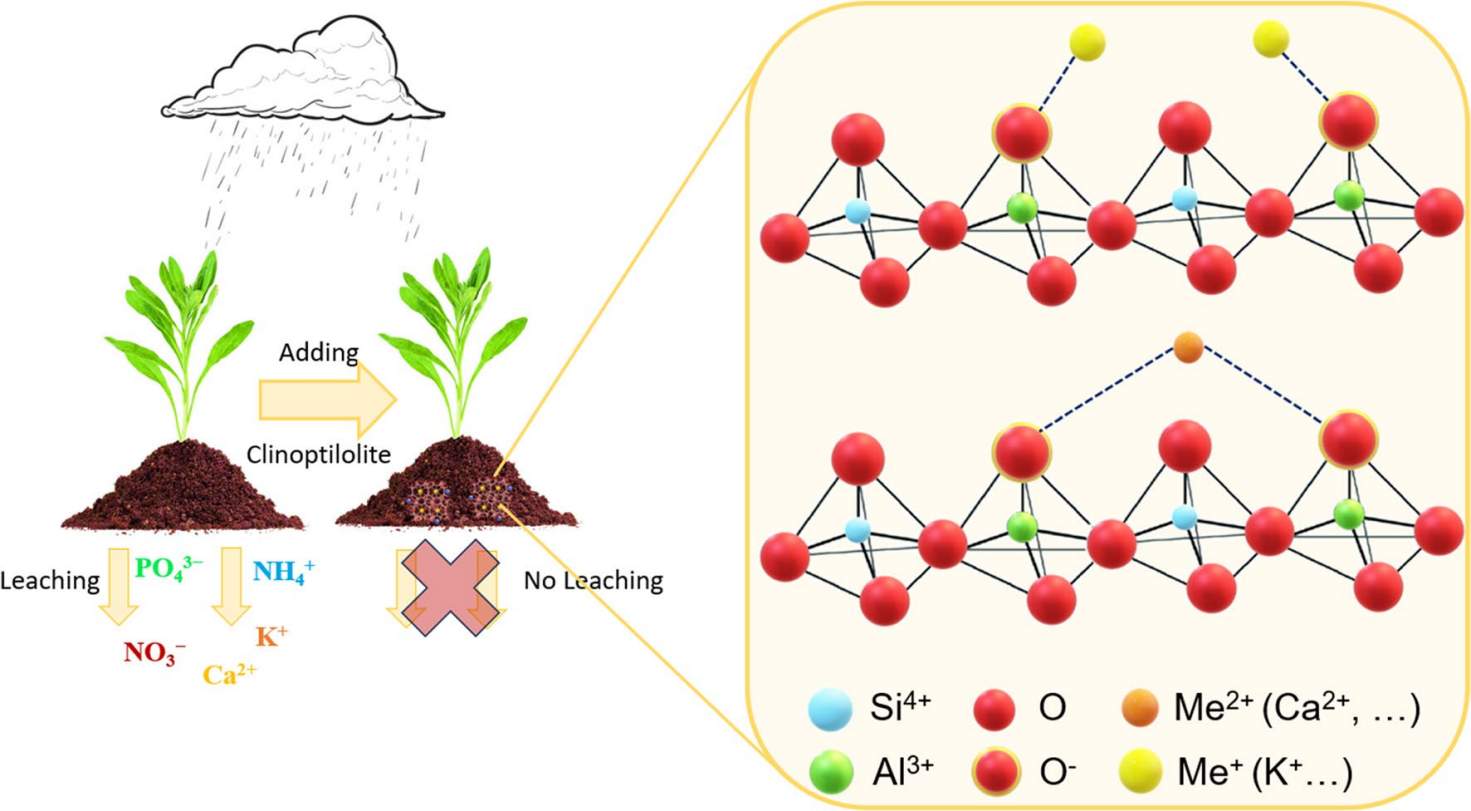
Use of clinoptilolite in agriculture. Adapted from Mondal et al. (2021)

Thanks to its adsorption capacity and ion exchange properties, clinoptilolite is also used as fertilizer, additive, or as a carrier of agrochemicals, pesticides, insecticides, antibacterial agents, and growth stimulators in agriculture (Matsumura et al. 2003; Rehakova et al. 2004). In fact, as schematize in Fig. 18, generally, natural and surface-modified zeolites can efficiently hold water and nutrients including ammonium (NH_4_^+^), nitrate (NO_3_^−^) and phosphate (PO_4_^3−^), potassium (K^+^), and so on in their porous structures. Therefore, zeolite application, and among them clinoptilolite, can improve both water use efficiency and nutrient use efficiency (NUE) in agricultural activities and consequently can reduce the potential for surface and groundwater pollution (Haemmerle and Tschegg 2023; Mondal et al. 2021; Nakhli et al. 2017).

In this context, the zeodratation capacity of the clinoptilolite is crucial since it may be used to improve the water balance in the soil, especially during the growth process of moisture-sensitive vegetables, special vineyard cultures, and fruit. Moreover, it can be added that zeolite’s effectiveness against insects relies on their capacity to remove the insects’ outer cuticle, through abrasion or through adsorption of epicuticular lipids. The result of both these processes is a rapid water loss from the insect’s body which leads to the insect’s death due to desiccation (De Smedt et al. 2015). Sang-Kyu et al. (Kam et al. 2002) investigated the adsorption and desorption of a particular pesticide, mainly triadimefon, by using clinoptilolite and compared its performance with the one achieved through a synthetic faujasite. They found out that the desorption of triadimefon occurred more easily by using the natural zeolite through four successive desorption cycles. In another study, Choo et al. (2022) found that the co-application of clinoptilolite and NPK fertilizers can be used to improve nitrogen availability and pineapple productivity in tropical peat soils. Furthermore, N-loaded clinoptilolite demonstrated its efficacy in raising soil inorganic N content, minimizing ammonia volatilization by 29% and 33% in the 2 years, thus reducing nitrogen loss but also preventing environmental pollution. N-loaded clinoptilolite also increased the soil NH_4_^+^-N and NO_3_^-^-N content during the tillering and grain-filling stages of rice, which in turn increased the rate of maximum N accumulation, shortened the duration, and promoted N accumulation and translocation in rice plants. The results confirmed that the use of this zeolite is an environmentally friendly and effective management model for on-farm practices (Sun et al. 2023).

Other applications in the agricultural field consist of moisture regulation of grain and cereal and in the capture of heavy metals such as Pb, Cd, Zn, and Cu, as discussed in the previous section. One more interesting use is as an additive for egg growth or bone meal together with its exploitation in pet bedding or cow’s feed in order to reduce the assimilation of nitrates deriving from the water thus lowering the concentration of aflatoxins in their milk (Rehakova et al. 2004).

In conclusion, clinoptilolite-based fertilizer has several advantages (Rehakova et al. 2004): (i) natural and non-toxic material, (ii) easily applied at the beginning of the vegetation period, (iii) beneficial effect through the years, (iv) ecologically advantageous, and (v) resistant to washing out caused by torrential rains. All these properties make this zeolite suitable for a more sustainable agriculture.

## Innovative and future applications

### Medical and health applications

**Fig. 19:**
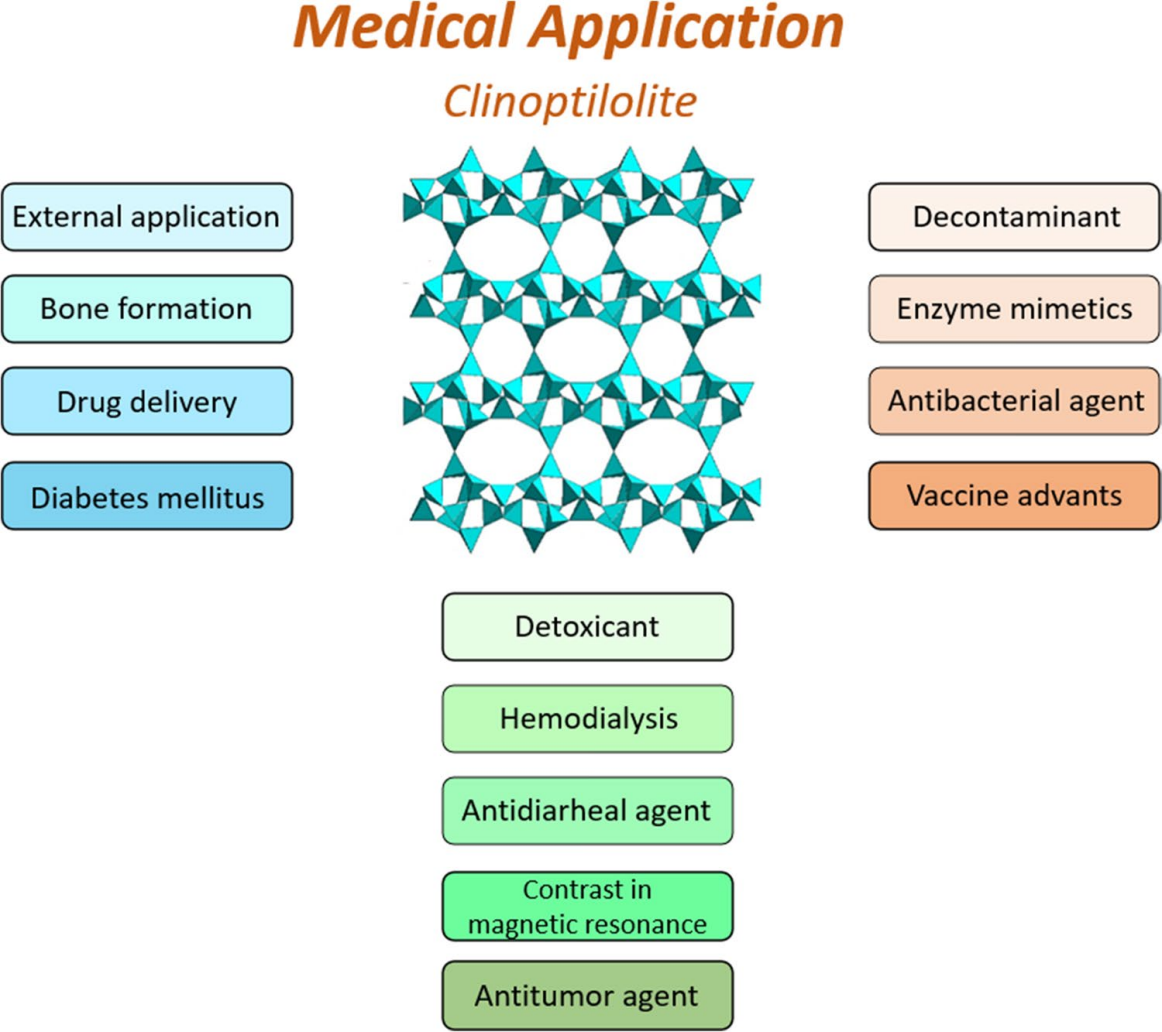
Different uses of clinoptilolite in medical applications

As extensively described in the previous sections, natural zeolites and, in particular, clinoptilolite are widely used in industry, agriculture, water treatment, and so on. However, recently, their use has been exploited in other, more specialized and innovative areas, i.e., biomedical and cosmetic applications (Hubner et al. 2020; Neag et al. 2022), which promotes the use of this natural material. It is noteworthy to remember that not all natural zeolites are safe for the human. In fact, erionite forms fragile wool-like fibers and exhibits properties similar to those of asbestos (Beaucham et al. 2018). On the other hand, clinoptilolite is considered safe and not toxic; thus, it is employed in some therapeutic applications (Kraljević Pavelić et al. 2018). Some uses of clinoptilolite in medical applications are illustrated in Fig. 19. In fact, biomedical processes are closely related to ion exchange, adsorption, and catalytic properties. Thus, it is evident that natural zeolites could be considered a promising solution in pharmaceutical industry and medical sector.

Regarding the application in the medical sector, it is worth mentioning that zeolites are characterized by well-known biological properties, and chemical and biological stability, as well as the fact that they reversibly bind small molecules, have shape and size selectivity, possess immunomodulatory activity, and, finally, can offer the possibility of metalloenzymatic mimicry (Ariton et al. 2022). The role of clinoptilolite has been recognized in zootechnics and veterinary medicine since it is able to improve the physical fitness and efficiency of farm animals by removing numerous harmful substances from the body including ammonia, nitrates, mycotoxins, and other toxins. Furthermore, this natural zeolite acts on intestinal lymphoid tissues with a positive impact on the intestinal ecosystem and boosts the immune system (Mastinu et al. 2019). Through in vitro and in vivo investigations, the use of clinoptilolite to treat mice and dogs with cancer was found to improve their general health, prolong life span, and reduce tumor size in particular cases. An immunostimulant effect of this natural zeolite was noted, which could be considered the main mechanism of its anti-metastatic ability (Zarkovic et al. 2003). In a recent study (Paryzhak et al. 2023), the immunostimulatory effects of clinoptilolite, eventually doped with silver, were investigated and it was observed an increase in the humoral immune response, acting in vivo as an adjuvant and leading to the stimulation of antibody-producing plasma cells, demonstrating that clinoptilolite acts in vitro on neutrophil granulocytes with their further activation. The obtained results suggested a possibility for its use in medicine as modulator of the immune response and antifungal remedies.

Clinoptilolite is also employed as detoxifying agent. For the first time in 2012, Beltcheva et al. (2012) studied its efficacy in lead poisoned mice and they observed that the natural zeolite caused a reduction of levels of lead’s accumulation in the intestine and provided the brain tissue with a protective effect. In addition, clinoptilolite was proven to restore cholinesterase activity at the level system in rats that showed intoxication derived from organophosphates (Nikpey et al. 2013). Raj et al. (2021) tested the ability of this natural zeolite to bind mycotoxins in order to prevent the gastrointestinal absorption of aflatoxin B1 and ochratoxin A and its effects on the health status and performance parameters of broilers. The results demonstrated the improvements in productive performance and reduction of mycotoxin residue levels in tissues, validating a beneficial effect of clinoptilolite. In another study, the role of this mineral was tested to contrast the toxicity of Cd and Pb in small rodents’ organisms, resulting a successful detoxifier against heavy metals and other toxic elements in living organisms (Beltcheva et al. 2022).

Moreover, this natural zeolite has proven to be effective in ammonia detoxification effect (Beigbeder 2023). It is known that ammonia is a waste product of protein metabolism, which is then converted into urea and expelled from the body through kidneys. To avoid an excess of this molecule and restore the integrity of the intestinal barrier thus alleviating side effects like nausea and diarrhea, the use of clinoptilolite could be a possible solution (Lamprecht et al. 2015). However, few studies were conducted on humans; therefore, further investigations are required for human use.

Finally, it is crucial to mention that in the medical field, different clinoptilolite processing procedures may cause substantial changes in the physical-chemical properties of the material, affecting biological properties as well. Thus, a team of researchers recently investigated different clinoptilolite materials obtained by various production methods and evaluated them for detoxification properties in vitro and in AlCl_3_^−^-intoxicated rats in vivo (Kraljević Pavelić et al. 2017). The results demonstrated no aluminum leakage of these materials into the blood or organs of tested animals, proving for the first time the efficiency of clinoptilolite in the detoxification of aluminum in vivo, and providing scientific data on safety issues and usage for detoxification purposes.

It is also noteworthy to cite the antioxidant effect of this natural zeolite. Saribeyoglu et al. (2011) studied the ability of a cell to maintain functional homeostasis which depends on protective antioxidant enzymes, such as superoxide dismutase (SOD) and glutathione (GSH) which protect cells against oxygen radicals and toxic compounds. If their levels are low, an excess of reactive oxygen species (ROS) is recorded, and they can damage the DNA, proteins, and lipids. This process is called oxidative stress and is involved in various diseases including obesity, atherosclerosis, neurological diseases, and cancer. The authors of this study showed in vivo antioxidant properties of clinoptilolite against hepatectomy-induced oxidative stress in rats, observing a decrease of oxidant injury and an increase of antioxidant capacity after partial hepatectomy, showing nontoxic and beneficial effects of clinoptilolite on the remnant liver. However, the exact antioxidant mechanisms of clinoptilolite are not well defined and further studies are required in order to assess the role of this natural material in clinical field to treat various pathologic conditions.

Finally, a further study successfully demonstrated the use of clinoptilolite in the management of d-glucose in subjects with impaired glucose metabolism or diabetes (Markoska et al. 2023). This field of application is particularly interesting in the context of a growing interest in integrative medicine approaches based on the use of different natural compounds due to their high biocompatibility and very low toxicity. The presented study provided new insights into sugar-zeolite clinoptilolite interactions for researchers in the field. The data presented deserve further investigation, as the material clearly shows potential in the management of hyperglycemia, e.g., in obese people, diabetics, and people with metabolic syndrome, where it could help regulate blood glucose levels. Finally, it is important to state that the quality and chemical composition of clinoptilolite zeolite materials are a prerequisite for in vivo applications. It is also interesting to highlight the possible antibacterial and antiviral effects of clinoptilolite. For instance, different studies in vivo reported the capability of this zeolite to increase the antimicrobial resistance toward *E. coli*, observing a decrease of the microbial population in the intestine (Jahanbakhsh et al. 2015). Similarly, the antiviral properties of clinoptilolite were investigated by Grce and Pavelić (2005) on different kinds of human viruses. Specifically, they performed in vitro experiments on human adenovirus 5, herpes simplex virus type 1 (HSV1), human enteroviruses coxsackievirus B5, and echovirus 7. The results demonstrated a significant inhibitory effect upon viral proliferation. Precisely, the inhibition of HSV1, coxsackievirus B5, and echovirus 7 was more efficient than that of adenovirus 5. The authors hypnotized that antiviral effect of clinoptilolite could be non-specific and could be due to the incorporation of viral particles into pores of the zeolite. Such inactivation of viral particles by natural zeolite would be extremely interesting for viruses that infect the digestive tract. Moreover, since clinoptilolite can be orally administrated without toxicity, it could be used for different therapeutic purposes.

**Fig. 20:**
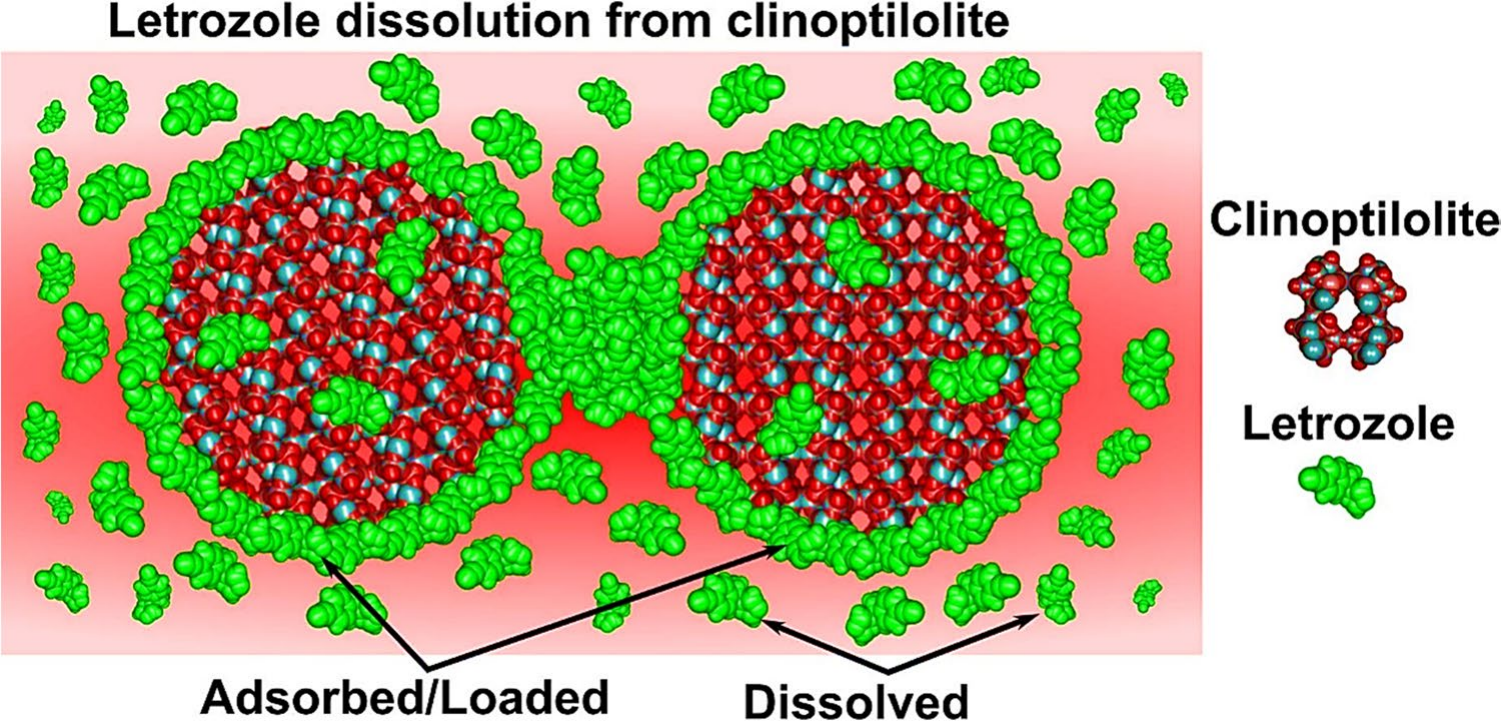
Representation of drug delivery system for the dissolution of letrozole anticancer drug. Adapted from (Kukobat et al. 2024) with the permission of Elsevier

To conclude, it is remarkable to consider clinoptilolite as a potential drug deliverable. For instance, in 2014, Tondar et al. (2014) proposed it as a non-toxic, highly available, and low-cost microporous material for oral drug delivery of aspirin. Recently, another research team reported the use of this zeolite for the high dissolution of directly adsorbed anticancer drugs, mainly letrozole (LTZ), for the development of drug delivery systems with high bioavailability (Kukobat et al. 2024); see Fig. 20. They demonstrated that the in vitro dissolution of this drug reached 95% after 23 h in acidic environment, which was faster than the dissolution of pure LTZ molecules. The main advantage of this system is its rapid onset of action and high bioavailability. This work demonstrates the possibility of improving the dissolution of poorly soluble LTZ, which is promising for the further development of drug delivery systems.

Finally, it is noteworthy to mention a very recent study in the field of nanomedicine conducted by Segneanu et al. (2024). The main aim was to combine the power of medicinal plants with nanotechnology to create advanced scaffolds that boast improved bioavailability, biodistribution, and controlled release. In this work, a new nanocarrier was obtained from *Sideritis scardica Griseb* and clinoptilolite, to reach prolonged action, vectorization, specificity, and superior therapeutic action. The study findings suggest the potential applications as a promising aspirant in neurodegenerative strategy. However, further research is needed to explore its potential biomedical applications.

### Applications of clinoptilolite in the food sector

For several years, clinoptilolite has been employed as an additive in animal’s feed because of its ability to adsorb toxins generated by molds and parasites, thus providing the animals with a better adsorption of macro and micro-nutrients. Recently, scientific progress has made the use of this material in human’s food sector possible (Deshmukh et al. 2023; Singh and Kumar 2023; Tzia and Zorpas 2012; Villa et al. 2022).

Surprisingly, clinoptilolite in the purified form was successfully tested for the adsorption of gluten derived from different origins, i.e., wheat, barley, and rye (Ranftler et al. 2022). Tests were performed in vitro, in artificial gastric and intestinal fluids, and the adsorption capacity was determined via a certified validated method (ELISA). An in vitro study regarding the binding capacity of gluten onto purified clinoptilolite tuff is available in the literature. The idea of this unconventional utilization of clinoptilolite came from the necessity of finding innovative solutions to numerous gluten-related diseases, whose incidence has grown rapidly in the last few years. This result could provide the basis for a future study in humans. Another interesting application of this material concerns beer filtration (Cadar et al. 2023). It is well known that the clarity of this beverage is essential to its marketability and good consumer approval. Thus, during the filtration step, many unwanted constituents that cause haze formation in beer are removed. In this context, clinoptilolite was efficiently used as natural and sustainable filter. The results showed that the taste, flavor, and pH of the filtered beer were generally not affected by filtration, while turbidity and color decreased with an increase in the zeolite content used in the filtration. The results showed that natural zeolites could be considered promising aids for beer filtration and can be easily adopted without significant changes in brewing equipment and preparation protocols in the beer industry.

Another interesting food-related application concerns food packaging, where clinoptilolite is adopted as active packaging, to prolong the conservation or shelf life of the product, thus reducing food waste and economic loss due to the degradation of the food itself (Villa et al. 2022). Active packaging includes antimicrobials, ethylene adsorbents, or oxygen scavengers. In this context, different studies related to ethylene adsorption on natural zeolites, including clinoptilolite, were reported in the literature (Erdoğan et al. 2008; Vargas-Hernández et al. 2015). Ethylene belongs to the volatile organic compounds (VOCs) class. It is a natural hormone that explicates its regulatory function in the growth, development, and senescence of plants and horticultural products. However, an excess of this substance leads to ripening of fruits and vegetables, thus compromising their postharvest quality and shelf life. Natural zeolite, doped with copper and zinc cations, effectively excludes ethylene and delays the ripening of fruits, i.e., tomatoes. In fact, the use of zeolite has different effects on the gas permeation or adsorption through the packaging films which can control the degradative parameters of fruits and vegetables (Hosseinnia et al. 2024). This emerging technology allows to increase in the shelf life, thus increasing the market potential for fresh fruits and vegetables (Mariah et al. 2022). Recently, a new type of packaging, consisting of the combination of clinoptilolite (adsorbent) and a biopolymer, mainly chitosan (carrier), with the advantage of combining the adsorption capacity of the natural zeolite with the biodegradability and the antimicrobial properties of the biofilm support. The results showed that this composite film can effectively adsorb ethylene, varying the adsorption capacity depending on the zeolite amount or film drying process, with good potential for application on packaging and storage of fruits (do Nascimento Sousa et al. 2020). Furthermore, it is noteworthy that clinoptilolite was also tested and recognized as safe by EFSA (European Food Safety Authority) to be incorporated in Fe-based oxygen adsorbers in which iron, which is the main active phase, reacts with oxygen, thereby removing it from the primary packaging thanks to the formation of iron oxide and hydroxide. These oxygen scavengers are designed for long-term storage conditions at variable temperature and suitable for dry foods like bakery products, tea, or coffee; however, the direct contact with liquids is not possible at the moment (EFSA Panel on Food Contact Materials Flavourings and Processing Aids (CEF) 2013). Moreover, the role of clinoptilolite was investigated as an antimicrobial agent in food packaging, since the growth of microorganisms makes the food harmful for human health as well as organoleptically unacceptable for consumption. Kuley et al. (2012) examined the effect of different doses of this natural zeolite in vacuum-packaged sardine fillets. The results demonstrated that clinoptilolite is a inexpensive and efficient solution not only for removing off undesirable odor but also for the deletion of ammonia and biogenic amine accumulation, thus increasing the expected shelf life and improving sensory quality.

Finally, the influence of natural clinoptilolite was investigated to prevent the formation of molecules that can degrade the food product. In this context, a study was carried out studying the effect of the zeolite on ammonia, cadaverine, and other polyamines produced by different microorganisms (i.e., *Staphylococcus aureus*, *Escherichia coli*, *Pseudomonas aeruginosa*, *Listeria monocytogenes*, and *Salmonella paratyphi* A, and so on). The obtained results could be used in the food sectors to prevent undesirable compound production by pathogenic bacteria which constitute a risk to the consumers’ health and can cause several food-borne diseases (Özogul et al. 2016).

### Applications of clinoptilolite for clean energy production

**Fig. 21:**
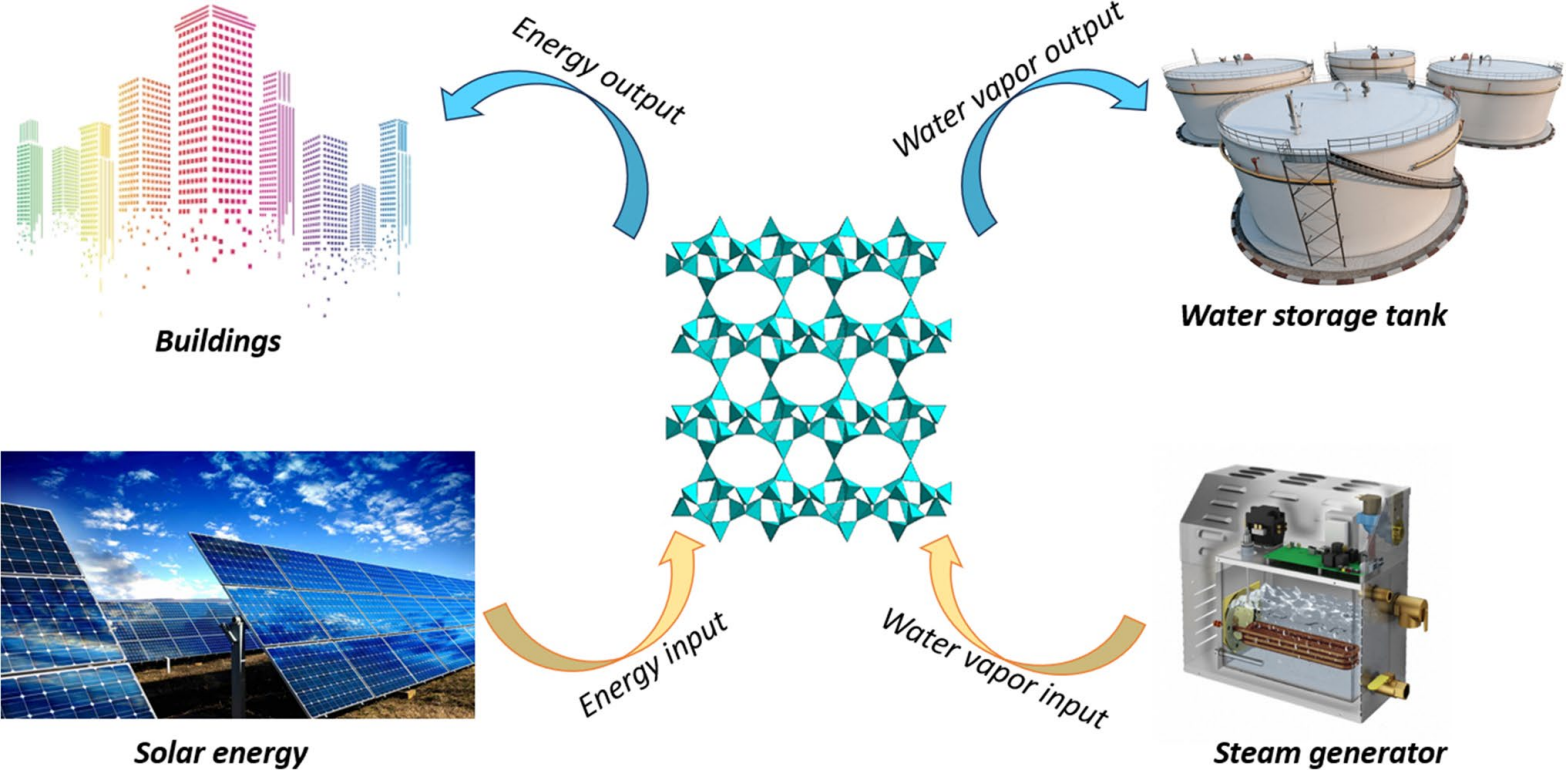
Scheme of Thermal Energy Storage (TES) System based on zeolite

It is well known that a major part of the energy demand is for heating water and rooms. For centuries, fossil fuels have been widely used to produce thermal energy from their combustion, but, due to the high emissions of pollutants resulting, more sustainable solutions are being sought to limit emissions and environmental pollution. Another drawback is the long-term storage due to the numerous heat losses involved. Therefore, the main challenge of this century consists of the development of energy from renewable sources, coupled with a reduction in energy waste. To overcome these disadvantages, many studies focused attention on more sustainable alternative materials (de Gennaro et al. 2022; Kouchachvili et al. 2023). Among them, zeolites appear suitable for this purpose since they are able to store and release thermal energy through cycles of hydration and dehydration and they can reduce CO_2_ emissions (Breck 1973; Ristić 2022). Some researchers focused on the possibility of storing waste heat energy on the zeolite’s framework, even for a long period of time, with the aim of releasing it subsequently, when it is needed (Carotenuto 2023). This is the idea behind Thermal Energy Storage systems (named TES). A schematic representation of TES based on zeolites is reported in Fig. 21.

Depending on the “nature” of stored heat (mainly sensible, latent or thermochemical), TES systems can be subdivided into three main categories. Zeolites seem to be the perfect candidates as adsorbents for TES systems because of their hydrophilic properties and their ability to store a huge amount of heat, which is a heat of adsorption (Nasruddin et al. 2020). Additionally, it should be emphasized that both synthetic and natural zeolites, i.e., 13X and clinoptilolite, respectively, have been investigated for this application. During these studies, several properties have been compared, including zeolite weight loss, water adsorption kinetics, water adsorption isotherm, thermal stability, porosity, and thermal conductivity.

For instance, De Gennaro et al. (2022) compared the performance of 13X with those of clinoptilolite-based material, and chabazite-based volcanic rock (both materials contained 50–60% of zeolite). By considering the weight loss, which is an important parameter for understanding the capability of zeolite to adsorb and later desorb water in the various cycles, they observed that it was doubled in 13X compared to clinoptilolite or chabazite and grew linearly with activation temperature. Regarding the adsorption kinetics of the various materials after heat treatment with various activation temperatures, it was found that chabazite took 170 and 220 min to reach 80% and 100% hydration respectively, clinoptilolite-based material took 300 and 540 min respectively, while 13X this occurred after 250 and 750 min. As far as thermal stability is concerned, it was noted that both natural and synthetic zeolites preserved their characteristics after numerous charge and discharge cycles. In conclusion, even though natural zeolites showed half the adsorption capacity of 13X, they resulted thermally stable, less expensive, largely available, and faster to reach equilibrium conditions than zeolite 13X.

Nasruddin et al. (2020) carried out a study on the performance of a natural Indonesian zeolite as a potential adsorbent for thermal storage. They observed a temperature lift of 50°C and an energy storage density of 63.94 kW/m^3^, which is lower than the one obtained with synthetic zeolite 13X or alumina but higher than the one achievable with water or silica gel. This study confirmed the necessity to further investigate the role of clinoptilolite for energy applications on a large scale, since this mineral showed great potential but there is the need to enhance the adsorption capacity through the activation and modification of this material.

As a whole, although 13 X is the most used zeolite in TES systems because of its high performance, clinoptilolite could represent a valid alternative, since it is cost-effective, it is largely available in nature, and, despite the lower water adsorption capacity, it showed faster kinetics and a not negligible energy density, greater than the one achievable with silica gel and water. Other investigations are necessary to enhance the properties of this natural zeolite, for example by introducing extra-framework cations and evaluating the properties in order to make it competitive with 13X.

Remaining in the field of heat storage, another study conducted by Akgün and Yılmaz (2019) investigated the possibility of blending cement with clinoptilolite for energy-efficient building design. Precisely, the main aim was the rational and sustainable use of renewable energy sources, i.e., solar energy, which can be stored and used when it is necessary. The main idea was to collect solar heat during the day by the wall and release it at night. To do this, the researchers blended the cement with different amounts of clinoptilolite (10, 30, 50%), which presents a high heat storage capacity. The results demonstrated an improvement in the thermal insulation and heat storage ability of mortars containing zeolite-blended cement. Precisely, the mixture containing 10% of clinoptilolite exhibited the highest heat capacity (1.93 J (m^3^K)^−1^) and the lowest thermal diffusivity coefficient (1.409 m^2^s^−1^). This means that most of the heat is absorbed by the material and very little fraction is transmitted. Thus, these findings could lead to some advantages in terms of heat storage, promoting the use of a natural material. Moreover, in a recent study (Lizcano et al. 2021), some researchers proposed the use of clinoptilolite as a microwave-susceptible material. In fact, microwave heating systems are more specific and efficient heating systems since they do not depend on the material’s thermal conductivity. Due to the high-temperature resistance of this zeolite, it was proposed as a candidate for this kind of system. Of course, the ability to interact with microwave radiation depends on its composition and structure. The results of the experiments highlighted that clinoptilolite was able to reach 65°C, 75°C, 82°C, and 110°C after 15, 30, 45, and 60 s of exposure to microwave radiation. The results obtained suggested the possibility of using this zeolite as a possible candidate microwave susceptor, but further studies are required.

Finally, it is noteworthy to mention the exploitation of clinoptilolite in proton exchange membrane fuel cells (PEMFC) to produce clean energy. In fact, this technology represents a promising strategy to overcome the reliance on fossil fuels since the residue of the fuel cell is water and heat. In this context, Romero et al. (Baleón-Romero et al. 2022) investigated the role of clinoptilolite as an alternative to platinum-based catalyst (expensive and inclined to rapid deactivation) to produce energy in a fuel cell where bioethanol was used as fuel. The natural zeolite, in combination with carbon and hydrogel as a solid electrolyte, showed a great performance. The findings revealed that the maximum power by the cell was 0.005892 mV in 900 mm^2^. This performance was attributed to the presence of different cations in the zeolite’s structure, such as Ca, Mg, Si, Al, K, and Fe. In fact, some of these can act as co-supports whereas others act as an active phase, making the oxidation of bioethanol in the anode possible and, in the meanwhile, allowing the oxygen reduction in the cathode. This result confirms the great potential of the clinoptilolite also in this field; however, further research is encouraged to increase the efficiency.

## Conclusions

In this study, a comprehensive overview of the structure and key properties of natural zeolite clinoptilolite is reported. This material possesses remarkable attributes that render it suitable for diverse uses. Our aim is to contribute to and, more importantly, advocate for the adoption of sustainable, non-toxic, biocompatible, and cost-effective materials, minimizing environmental impact and leveraging natural resources. By reducing reliance on materials associated with significant pollution during production, we aim to encourage the utilization of what nature already provides. The focus of our endeavor is to shed light on the potential applications of a widely abundant zeolite, clinoptilolite, in unconventional and relatively unexplored domains. This zeolite shows promise in various fields, including agriculture, pollution control, drug delivery, and energy production. Clearly, additional research and investigations are imperative to comprehend underlying mechanisms and expand applications, particularly through in vivo studies for medical applications. In essence, this review serves as a resource intended to promote further research and development, acting as a springboard for advancements in the understanding and utilization of clinoptilolite, a sustainable material for many applications.

## Data Availability

All data generated or analyzed during this study are included in this published article.
